# Reversible Encapsulation of Xenon and CH_2_Cl_2_ in a Solid‐State Molecular Organometallic Framework (Guest@SMOM)

**DOI:** 10.1002/anie.201910539

**Published:** 2019-10-11

**Authors:** Antonio J. Martínez‐Martínez, Nicholas H. Rees, Andrew S. Weller

**Affiliations:** ^1^ Chemistry Research Laboratories Department of Chemistry University of Oxford Oxford OX1 3TA UK; ^2^ Current Address: CIQSO-Centre for Research in Sustainable Chemistry and Department of Chemistry University of Huelva Campus El Carmen 21007 Huelva Spain

**Keywords:** encapsulation, rhodium, single-crystal to single-crystal, solid-state chemistry, xenon

## Abstract

Reversible encapsulation of CH_2_Cl_2_ or Xe in a non‐porous solid‐state molecular organometallic framework of [Rh(Cy_2_PCH_2_PCy_2_)(NBD)][BAr^F^_4_] occurs in single‐crystal to single‐crystal transformations. These processes are probed by solid‐state NMR spectroscopy, including ^129^Xe SSNMR. Non‐covalent interactions with the ‐CF_3_ groups, and hydrophobic channels formed, of [BAr^F^_4_]^−^ anions are shown to be important, and thus have similarity to the transport of substrates and products to and from the active site in metalloenzymes.

## Graphical abstract

**Access control**: Reversible encapsulation of CH_2_Cl_2_ or Xe in a non‐porous solid‐state molecular organometallic (SMOM) framework of [Rh(Cy_2_PCH_2_PCy_2_)(NBD)][BAr^F^_4_] occurs in single‐crystal to single‐crystal transformations. The process is similar to the transport of substrates and products to and from the active site in metalloenzymes.

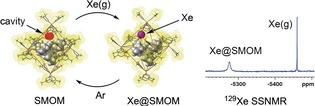

Solid‐state molecular organometallic chemistry (SMOM‐Chem)^1^ offers opportunities in synthesis and catalysis using well‐defined organometallic species in single‐crystal to single‐crystal (SC–SC) transformations.^2^ For example, the isolation and characterization of σ‐alkane complexes in the solid state is achieved by a simple solid/gas SC–SC hydrogenation reaction of an alkene precursor such as [Rh(Cy_2_PCH_2_CH_2_PCy_2_)(NBD)][BAr^F^_4_] (NBD=norbornadiene, Ar^F^=3,5‐(CF_3_)_2_C_6_H_3_) to form the corresponding σ‐alkane complex, **[1‐NBA][BAr^F^**_**4**_ **]** (Figure 1; norbornane=NBA).^3^ Further examples of NBA,^4^ pentane,^5^ cyclooctane,^6^ isobutane and cyclohexane^7^ σ‐complexes have all been reported with a variety of [Rh(chelating phosphine)]^+^ ligand sets, and some of these show remarkable stability in the solid state (months at 298 K). The stability of these SMOM systems allows for these σ‐complexes to undergo further SC–SC transformations. For example, reaction with D_2_ (leading to C−H/C−D exchange at the alkane),^7^, ^8^ H_2_ loss (acceptorless alkane dehydrogenation)^7^ or substitution of the NBA ligand, e.g., Figure 1 B.^1^, ^7^ Key to this reactivity is the well‐defined confined microenvironment provided in the solid state by the [BAr^F^_4_]^−^ anions that provide a relatively robust encapsulating framework—related to MOFs^9^ or supramolecular catalysts.^10^ This supports the structural reorganization associated with the reactive metal center and also allows reversible access for gases and small organic compounds, albeit in what is essentially a non‐porous material.^1^ That σ‐alkane complexes are unstable and transient in solution, even at low temperature,^11^ demonstrates the stabilizing effect of the non‐covalent anion microenvironment.

**Figure 1:**
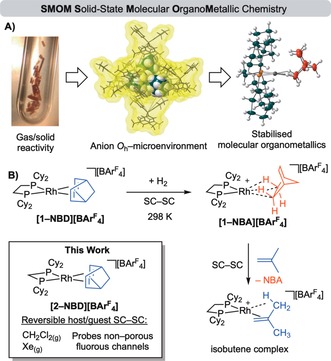
A) The SMOM methodology. B) Solid/gas synthesis of a σ‐alkane complex **[1‐NBA][BAr^F^**_**4**_ **]** and onward reactivity via sequential single‐crystal to single‐crystal (SC–SC) transformations.

While these systems also promote catalysis (e.g. 1‐butene isomerization) this may occur at, or close to, the crystal surface.^1^, ^12^ A key question, then, is how substrate/product molecules move in and out of the crystalline lattice on the timescale of synthesis (minutes to hours). Brammer and co‐workers have reported reversible SC–SC uptake of alcohols in non‐porous coordination polymers [Ag_4_(O_2_C(CF_2_)_2_CF_3_)_4_(TMP)]_*n*_ (TMP=tetramethylpyrazine) and suggested that interdigitated fluorous groups provide a mechanism for transport through the crystal.^13^ We now show that by using [Rh(Cy_2_PCH_2_PCy_2_)(NBD)][BAr^F^_4_] (**[2‐NBD][BAr^F^**_**4**_ **]**) reversible uptake and release of CH_2_Cl_2_ vapor and Xe gas occurs in a SC–SC manner to form non‐covalently bound host–guest complexes in a well‐defined metal‐localised cavity, via the hydrophobic fluorous channels of the CF_3_ groups of the [BAr^F^_4_] anions.

Addition of NBD to [Rh(Cy_2_PCH_2_PCy_2_)(1,2‐F_2_C_6_H_4_)][BAr^F^_4_]^14^ and crystallisation from a CH_2_Cl_2_/pentane mixture yielded orange prismatic crystals (86 % yield). Single‐crystal X‐ray diffraction, elemental analysis, solution and solid‐state NMR (SSNMR) spectroscopic data confirmed the formulation as a diene complex [Rh(Cy_2_PCH_2_PCy_2_)(η^2^η^2^‐NBD)][(CH_2_Cl_2_)_0.75_⊂BAr^F^_4_] (**[2‐NBD][(CH_2_Cl_2_)_0.75_**⊂**BAr^F^**_**4**_ **]**).^15^ The solid‐state molecular structure (*R1*=4.4 %) shows that the Rh cation is located inside an ≈*O*_h_ cage constructed of six [BAr^F^_4_]^−^ anions (Figures 2 and 3 A), alongside an encapsulated molecule of CH_2_Cl_2_ that sits between two [BAr^F^_4_]^−^ aryl rings and the Cy_2_PCH_2_PCy_2_ ligand, that is, CH_2_Cl_2_@SMOM. We have recently reported a related structure that shows an encapsulated cyclooctane molecule within the *O*_h_‐[BAr^F^_4_] cavity.^6^ The CH_2_Cl_2_ molecule refined to 75 % occupancy, is disordered over two positions (0.65:0.10),^15^ and is supported by ClCH_2_Cl⋅⋅⋅F_3_C [range 2.685(3)–3.127(2) Å, sum of van der Waals radii=3.28 Å^16^] and Cl_2_CH_2_⋅⋅⋅F_3_C [2.425(2)–3.035(4) Å] non‐covalent interactions (Figure S19,S20).^17^ The methylene hydrogens (calculated positions) of the major disordered component point towards the centers of two aryl rings of the [BAr^F^_4_]^−^ anion [2.62, 3.04 Å].

**Figure 2:**
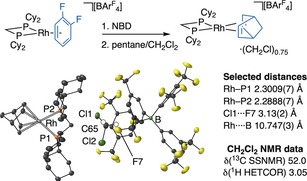
Synthesis of **[2‐NBD][CH_2_Cl_2_**⊂**BAr^F^**_**4**_ **]** and structure of the cation, proximal anion and confined CH_2_Cl_2_ (major component).

Consistent with the lack of crystallographically‐imposed symmetry in the cation, two distinct but broad resonances are observed in the 298 K ^31^P{^1^H} SSNMR spectrum [*δ* −24.6, *J*_RhP_≈120 Hz; −28.0]. In the ^13^C{^1^H} SSNMR spectrum notable resonances for the norbornene (*δ* 94.7, 91.7, 87.7 and 87.1) are observed, along with a single sharp resonance for the encapsulated CH_2_Cl_2_ at *δ* 52.0. This resonance also shows a cross peak at *δ* 3.05 in the ^1^H projection of the ^13^C/^1^H FSLG HETCOR SSNMR^18^ spectrum (Figure S7). This is significantly shifted from that in solution (*δ* 5.33) reflecting ring current effects from the proximal [BAr^F^_4_]^−^ anions, as we have noted previously for σ‐alkane complexes such as **[1‐NBA][BAr^F^**_**4**_ **]**.^3^, ^8^ The solution NMR data of dissolved crystals are unremarkable, save for a sharp singlet observed at *δ*(^1^H) 5.33 (≈1.5 H relative to the [BAr^F^_4_]^−^ anion) assigned to CH_2_Cl_2_, consistent with its 0.75 occupancy in the crystalline lattice. The single resonance (Figure S6) observed for the CH_2_Cl_2_ in the ^13^C{^1^H} SSNMR spectrum suggests dynamic disorder in the solid state.

When single crystals of **[2‐NBD][(CH_2_Cl_2_)_0.75_**⊂**BAr^F^**_**4**_ **]** are placed under dynamic vacuum (10^−2^ mbar) for 24 hours at 298 K, loss of the encapsulated CH_2_Cl_2_ molecule occurs to form **[2‐NBD][BAr^F^**_**4**_ **]** via a SC–SC transformation. The solid‐state structure (*R1*=4.0 %) shows essentially unchanged cation and anion structural units (Figure 3, A → B). However, the loss of CH_2_Cl_2_ (van der Waals volume=57 Å^3^) creates a hydrophobic cavity of ≈115 Å^3^ located inside the cage and coincident with the position of the CH_2_Cl_2_ molecule in **[2‐NBD][(CH_2_Cl_2_)_0.75_**⊂**BAr^F^**_**4**_ **]**. Such a cavity is absent in **[1‐NBD][BAr^F^**_**4**_ **]** reflecting the different steric requirements of Cy_2_PCH_2_CH_2_PCy_2_ and Cy_2_PCH_2_PCy_2_. *V* ${}_{\mathrm{CH}{}_{2}\mathrm{Cl}{}_{2}}$ /*V*_cavity_=0.50, which is within the limits defined by Rebek for the most effective host–guest interactions (0.55±0.09).^19^ There is a small (2 %) contraction of the unit cell volume on loss of CH_2_Cl_2_, reflected by a small compression of Rh⋅⋅⋅B distances from 10.747(3) to 10.531(2) Å respectively. Elemental analysis, ^13^C{^1^H} SSNMR and solution ^1^H NMR spectroscopy demonstrate loss of CH_2_Cl_2_ has occurred.^15^ Interestingly the ^31^P{^1^H} SSNMR spectrum now shows sharp signals at *δ* −23.3 and −27.1 in which both *J*_RhP_ and *J*_PP_ can be resolved (Figure S13). This transformation is reversible, and when crystals of **[2‐NBD][BAr^F^**_**4**_ **]** were exposed to CH_2_Cl_2_ vapor in an argon atmosphere for 48 hours **[2‐NBD][(CH_2_Cl_2_)_0.75_**⊂**BAr^F^**_**4**_ **]** reforms via a SC–SC gas/solid transformation as confirmed by single crystal X‐ray diffraction (*R1*=5.1 %) and ^31^P{^1^H} SSNMR spectroscopy.

**Figure 3:**
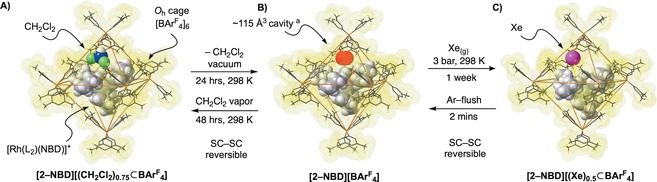
Synthesis and solid‐state structures of A) **[2‐NBD][(CH_2_Cl_2_)_0.75_⊂BAr^F^**_**4**_ **]**, B) **[2‐NBD][BAr^F^**_**4**_ **]** and C) **[2‐NBD][Xe_0.5_⊂BAr^F^**_**4**_ **]**, and reversible encapsulation of guest CH_2_Cl_2_ and Xe via gas/solid SC–SC transformations. L_2_=Cy_2_PCH_2_PCy_2_. Molecular structures show the host ≈*O*_h_‐[BAr^F^_4_] cages using van der Waals radii. [a] Cavity as calculated using the contact surface with Mercury CSD software package at a probe radius of 1.5 Å and the grid spacing 0.2 Å. See the Supporting Information for displacement ellipsoid plot and further details.

This reversible SC‐SC process with CH_2_Cl_2_ led us to consider whether the cavity in **[2‐NBD][BAr^F^**_**4**_ **]** could accommodate Xenon (van der Waals volume=50 Å^3^,^20^ *V*_Xe_/*V*_cavity_=0.43). Xenon finds application in structural biology as a probe for solvent and gas channels in metalloenzymes, due to its high atomic number and hydrophobicity.^21^ It also shows binding affinity in supramolecular cages,^22^ oxide frameworks,^23^ MOFs,^24^ cryptophanes,^25^ and porous coordination‐complex salts;^26^ and has been widely used as an NMR probe for the determination of pore size in framework materials,^27^ due to the sensitivity of δ(^129^Xe) to its local environment.^28^

When a crystalline sample of **[2‐NBD][BAr^F^**_**4**_ **]** was pressurized with Xe_(g)_ in a solid/gas reaction (3 bar, 298 K) for 1 day, no measurable change was observed by single‐crystal X‐ray diffraction. However, after one week a new, Xe@SMOM, product is formed, [Rh(Cy_2_PCH_2_PCy_2_)(NBD)][(Xe)_0.5_⊂BAr^F^_4_] **[2‐NBD][(Xe)_0.5_**⊂**BAr^F^**_**4**_ **]** via a SC–SC transformation. Analysis by single‐crystal X‐ray diffraction (Figure 4, *R1*=5.2 %) shows the Xe atom filling the cavity in the lattice of **[2‐NBD][BAr^F^**_**4**_ **]**, with a freely‐refined occupancy of 0.5. The overall reaction from **[2‐NBD][(CH_2_Cl_2_)_0.75_**⊂**BAr^F^**_**4**_ **]** to **[2‐NBD][(Xe)_0.5_**⊂**BAr^F^**_**4**_ **]** involves two consecutive SC–SC transformations (Figure 3 A→C). The structural metrics for the cationic [Rh(Cy_2_PCH_2_PCy_2_)(NBD)]^+^ unit do not change significantly. Pressurising **[2‐NBD][BAr^F^**_**4**_ **]** with Xe_(g)_ (3 bars, 298 K) for 3 weeks did not increase the proportion of confined Xe, suggesting either kinetic (pore blocking) or thermodynamic (equilibrium) conditions. The encapsulation of Xe produces only a small (≈1 %) expansion of the crystal cell volume from **[2‐NBD][BAr^F^**_**4**_ **]** [Rh⋅⋅⋅⋅B, 10.654(3) Å].

**Figure 4:**
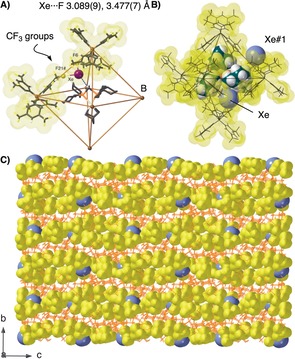
A) **[2‐NBD][(Xe)_0.5_**⊂**BAr^F^**_**4**_ **]** showing location of the Xe atom in the cage framework. Non‐interacting BAr^F^_4_^−^ anions are omitted, Xe is pictured as a ball, and [BAr^F^_4_]^−^ anions are shown with a van der Waals surface. B) *O_h_* [BAr^F^_4_]^−^ cavity (van der Waals surface), cation and Xe (van der Waals radii). Xe and Xe#1 are symmetry related, placed to show the relationship between neighboring *O_h_* units. C) Extend packing diagram highlighting the CF_3_ groups and Xe atoms (van der Waals radii).

The Xe atom has a number of weak non‐covalent interactions: Xe⋅⋅⋅H from the proximal PC*H*_2_P, 2.976(1) Å, and Xe⋅⋅⋅F_3_C from [BAr^F^_4_]^−^, 3.089(9)–3.477(7) Å [sum of van der Waals radii=3.48 and 3.74 Å respectively^16^, ^20^]. Well‐defined Xe⋅⋅⋅F intermolecular contacts are rare. Examples include: [C_6_F_5_Xe][B(CF_3_)_4_]^−^ [Xe⋅⋅⋅F=2.913(4) Å],^29^ Xe(C_6_F_5_)_2_ [Xe⋅⋅⋅F 3.30(1)–3.536(9) Å].^30^

Figure 4 B shows that while the Xe atom sits in the cavity of [BAr^F^_4_]^−^ anion distant from the potential site of metal reactivity (i.e. NBD), a symmetry‐related Xe atom from an adjacent motif (Xe#1) lies close to this {Rh(NBD)} unit. This provides a clue as to how gases (e.g. H_2_/D_2_,^8^ hydrocarbons,^1^ CO^12^) are primed for reaction at the metal center in solid/gas SC–SC SMOM reactions. Moreover, inspection of the extended packing diagram of **[2‐NBD][(Xe)_0.5_**⊂**BAr^F^**_**4**_ **]** (Figure 4 C) reveals that the Xe atoms sit in hydrophobic channels formed by the CF_3_‐groups of the [BAr^F^_4_]^−^ anions. A similar relationship for CH_2_Cl_2_ occurs in **[2‐NBD][(CH_2_Cl_2_)_0.75_**⊂**BAr^F^**_**4**_ **]**. When coupled with the encapsulated microenvironment in which the Rh‐center sits, there is a remarkable similarity between these guest@SMOM systems the hydrophobic channels that direct substrates and products towards, and away from, the active sites in metalloenzymes such as soluble methane monooxygenase hydroxylase or hydrogenases21a–21c

The encapsulation of Xenon can also be followed by ^31^P{^1^H} and ^129^Xe SSNMR spectroscopy at 298 K. A freshly prepared sample of **[2‐NBD][(Xe)_0.5_**⊂**BAr^F^**_**4**_ **]** was packed under an atmosphere of Xe (atmospheric pressure). In the resulting ^31^P{^1^H} SSNMR spectrum two broad singlets were observed at *δ* −23.8 and −27.2. Definitive evidence for Xe‐encapsulation was provided by the ^129^Xe SSNMR spectrum in which a broad resonance is observed at *δ* −5460 (fwhm 720 Hz), alongside an sharp upfield signal assigned to Xe_(g)_ (*δ* −5275), Figure 5. This chemical shift difference (≈200 ppm) is similar to that observed for Xe absorbed in the pores of MOF‐type materials.^31^ No exchange between the Xe@SMOM and Xe_(g)_ was observed by ^129^Xe EXSY SSNMR spectroscopy (mixing times 1.2 s to 5 ms), and the signal does not sharpen on decoupling ^19^F. In the ^19^F{^1^H} SSNMR spectrum a broad singlet at *δ* −63.2 is observed for the CF_3_ groups, with no coupling to ^129^Xe observed. It is likely that rotation of the CF_3_ groups is fast on the NMR timescale.

**Figure 5:**
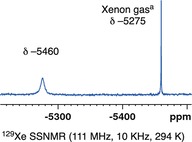
^129^Xe SSNMR NMR spectrum of **[2‐NBD][(Xe)_0.5_**⊂**BAr^F^**_**4**_ **]** referenced Xe_(g)_ at the zero pressure limit relative to O=XeF_4_ (see the Supporting Information).

Rapid loss of Xe from the crystal lattice was observed upon flushing the compound with argon gas at 298 K for 2 mins, that recovers **[2‐NBD][BAr^F^**_**4**_ **]** in a SC–SC transformation. Recharging with Xe gas (3 bar, 298 K, 1 week) retains crystallinity to give **[2‐NBD][(Xe)_0.5_**⊂**BAr^F^**_**4**_ **]** as shown by single‐crystal X‐ray crystallography, and ^31^P{^1^H} and ^129^Xe SSNMR spectroscopy.

In conclusion, we have demonstrated that reversible guest@SMOM binding can occur in [Rh(chelating‐phosphine)(NBD)][BAr^F^_4_] systems. Incorporation of CH_2_Cl_2_ or Xe in the non‐porous single crystalline lattice is facilitated by both non‐covalent interactions with the ‐CF_3_ groups of the [BAr^F^_4_]^−^ anions and the hydrophobic channels that these form in ensemble. This suggests viable pathways that allow the active SMOM metal centres, that are encapsulated in the anion microenvironments, to undergo SC–SC transformations in which simple, reactive, gases and hydrocarbons move in and out of the crystal lattice.^7^ The similarities with processes that occur in metalloenzymes, as probed by structural biology techniques, are particularly interesting. This suggests the possibility to exploit the benefits of the active sites in enzymes (microenvironment control of reactivity and selectivity^32^) with that of SMOM‐systems (controllable and precisely defined active metal–ligand sites) in solid/gas reactivity.

## Conflict of interest

The authors declare no conflict of interest.

## Supporting information

As a service to our authors and readers, this journal provides supporting information supplied by the authors. Such materials are peer reviewed and may be re‐organized for online delivery, but are not copy‐edited or typeset. Technical support issues arising from supporting information (other than missing files) should be addressed to the authors.

Supplementary
