# Supplementary material for: Reversible Encapsulation of Xenon and CH2Cl2 in a Solid‐State Molecular Organometallic Framework (Guest@SMOM)
Source: Angew Chem Int Ed Engl. 2019 Oct 11;58(47):16873–7. doi: 10.1002/anie.201910539 (PMC6899477; doi:10.1002/anie.201910539)
Supplement: Supplementary file 1 — Supplementary [file ANIE-58-16873-s001.pdf]

Angewandte Chemie
Eine Zeitschrift der Gesellschaft Deutscher Chemiker

Supporting Information

Reversible Encapsulation of Xenon and CH_2Cl_2 in a Solid-State Molecular Organometallic Framework (Guest@SMOM)

*Antonio J. Martínez-Martínez, Nicholas H. Rees, and Andrew S. Weller**

anie_201910539_sm_miscellaneous_information.pdf

Table of Contents

EXPERIMENTAL DETAILS 2
General methods and chemicals 2
Solution NMR spectroscopic data 2
Solid-state NMR spectroscopic data 2
Solid-state NMR samples preparation 3
Mass spectrometry data 3
SYNTHETHIC PROCEDURES AND CHARACTERIZATION DATA 4
Solution preparation and characterization of [Rh($Cy_2PCH_2PCy_2$)($\eta^2\eta^2$–NBD)][(CH_2Cl_2)$_{0.75}$⊂BAr$^F_4$] [2–NBD][(CH_2Cl_2)$_{0.75}$⊂BAr$^F_4$]. 4
NMR spectra of [2–NBD][(CH_2Cl_2)$_{0.75}$⊂BAr$^F_4$]. 5
Solid-state preparation and characterization of [Rh($Cy_2PCH_2PCy_2$)($\eta^2\eta^2$–NBD)][BAr$^F_4$] [2–NBD][BAr$^F_4$]. 9
NMR spectra of [2–NBD][BAr$^F_4$]. 10
Solid-state transformation of [2–NBD][BAr$^F_4$] into [2–NBD][(CH_2Cl_2)$_{0.75}$⊂BAr$^F_4$]. 13
Solid state preparation and characterization of [Rh($Cy_2PCH_2PCy_2$)($\eta^2\eta^2$–NBD)][(Xe)$_{0.5}$⊂BAr$^F_4$] [2–NBD][(Xe)$_{0.5}$⊂BAr$^F_4$] from [2–NBD][BAr$^F_4$]. 14
Solid-state transformation of [2–NBD][(Xe)$_{0.5}$⊂BAr$^F_4$] into [2–NBD][BAr$^F_4$]. 15
Solid state temporal studies of [2–NBD][BAr$^F_4$] xenon gas 15
Solid state recharging studies of [2–NBD][BAr$^F_4$] xenon gas ([2–NBD][BAr$^F_4$] ⇄ [2–NBD][(Xe)$_{0.5}$⊂BAr$^F_4$]) 15
NMR spectra of [2–NBD][(Xe)$_{0.5}$⊂BAr$^F_4$]. 16
X-RAY CRYSTALLOGRAPHY 18
Crystal structure determinations 18
Selected crystallographic and refinement data 18
X-ray crystal structure of [2–NBD][(CH_2Cl_2)$_{0.75}$⊂BAr$^F_4$] (CCDC 1946574) 18
X-ray crystal structure of [2–NBD][(BAr$^F_4$] (CCDC 1946575) 18
X-ray crystal structure of [2–NBD][(Xe)$_{0.5}$⊂BAr$^F_4$] (CCDC 1946576) 19
SUPPLEMENTARY X-RAY DATA 20
Molecular structure and metrics of [2–NBD][(CH_2Cl_2)$_{0.75}$⊂BAr$^F_4$] 20
Molecular structure and metrics of [2–NBD][(BAr$^F_4$] 22
Molecular structure and metrics of [2–NBD][(Xe)$_{0.5}$⊂BAr$^F_4$] 24
REFERENCES 26
AUTHOR CONTRIBUTIONS 26

EXPERIMENTAL DETAILS

General methods and chemicals

All manipulations (unless otherwise stated) were performed under an atmosphere of argon, using standard Schlenk techniques on a dual vacuum/inlet grease-free J. Young tap manifold. All new prepared and isolated organometallic compounds were stored in a MBraun glovebox under an atmosphere of argon. Glassware was dried in an oven at 140°C overnight prior to use. *n*-Pentane, *n*-hexane and dichloromethane (CH_2Cl_2, DCM) were all dried using an MBraun SPS-800 solvent purification system and degassed by three freeze-pump-thaw cycles. Deuterated dichloromethane (CD_2Cl_2) was purchased from Cambridge Isotope Laboratories Inc, dried by stirring over CaH_2 overnight before being vacuum distilled, subsequently degassed by three freeze-pump-thaw cycles and then stored over activated 3 Å molecular sieves. 1,2-difluorobenzene (1,2-$F_2C_6H_4$) was stirred over aluminium oxide (Al_2O_3) for two hours, filtered and then stirred over CaH_2 overnight before being vacuum distilled, subsequently degassed by three freeze-pump-thaw cycles and stored over 3 Å molecular sieves. $RhCl_3$·hydrate was purchased from Precious Metals Online PMO Pty Ltd and used as received. [Rh(COD)$_2$][BAr$^F_4$] was prepared as a red powder following literature methods.[1] Norbornadiene (bicyclo[2.2.1]hepta-2,5-diene, abbreviated as NBD) was purchased from Sigma-Aldrich, stirred with freshly cut small pieces of sodium metal for 24 hours and then vacuum distilled , subsequently degassed by three freeze-pump-thaw cycles and store over activated 3 Å molecular sieves. 1,5-cyclooctadiene (COD) was purchased from Aldrich and used as received. The chelating phosphine bis(dicyclohexylphosphino)methane (dcpm) was purchased from Aldrich, stored in a MBraun glovebox under an argon atmosphere and used as received. Sodium tetrakis[3,5-bis(trifluoromethyl)phenyl]borate[2] (NaBAr$^F_4$, Ar^F = 3,5-$(CF_3)_2C_6H_3$) was stored in a MBraun glovebox under an argon atmosphere and used as a crystalline solid. [Rh(Cy$_2$PCH$_2$PCy$_2$)($\eta^2\eta^2$–COD)][BArF$_4$] was prepared as a bright orange solid following literature methods.[3] High purity xenon gas (≥99.995) employed in the solid/gas transformations was purchase from Sigma-Aldrich. Xenon difluoride (XeF_2) was purchased from Sigma-Aldrich and used for standardisation and calibration of ^{129}Xe SS NMR spectroscopic experiments. All other chemicals were purchased from commercial sources and used as received. Elemental analyses were conducted by Mr Stephan Boyer at London Metropolitan University.

Solution NMR spectroscopic data

Solution NMR data were collected on the following NMR instruments with appropriate resonance frequencies: Bruker Avance III HD nanobay NMR spectrometer equipped with a 9.4 T magnet (^{1}H 400.2 MHz, ^{11}B 128.4 MHz, ^{13}C 100.6 MHz, ^{19}F 376.5 MHz, ^{31}P 162.0 MHz), a Bruker Avance III NMR spectrometer equipped with a 11.75 T magnet (^{1}H 499.9 MHz, ^{11}B 160.4 MHz, ^{13}C 125.7 MHz, ^{19}F 470.4 MHz, ^{31}P 202.4 MHz), and Bruker Avance NMR equipped with a 11.75 T magnet and a ^{13}C detect cryoprobe (^{1}H 500.3 MHz, ^{13}C 125.8 MHz). The temperature for NMR experiments was externally calibrated to observe the chemical-shift separation between the OH and CH_3 resonances in methanol. Non-deuterated solvents were locked to either standard external neat CD_2Cl_2 or 10% fluorobenzene in C_6D_6 samples. Residual protio solvent resonances were used as a reference for ^{1}H NMR spectra. ^{31}P{^{1}H} NMR spectra were referenced externally to 85% H_3PO_4 (D_2O). ^{19}F{^{1}H} NMR spectra were referenced externally to a 1% $CFCl_3$ sample in $CHCl_3$. ^{11}B{^{1}H} NMR spectra were referenced externally to a 5% $BF_3·OEt_2$ sample in C_6D_6. All chemical shifts (δ) are quoted in ppm and coupling constants (J) in Hz. In general, solution ^{19}F and ^{11}B NMR resonances for all the compounds reported are not included since they comprise of a single resonance at δ –62.9 and –6.5 respectively, corresponding the [BAr$^F_4$]$^-$ anion.

Solid-state NMR spectroscopic data

Solid-state ^{31}P{^{1}H} CP/MAS, ^{13}C{^{1}H} CP/MAS and fslg-HETCOR[4] NMR (SSNMR) spectra were collected on a Bruker Avance III HD spectrometer equipped with a 9.4 Tesla magnet, operating at 400 for ^{1}H, 101 MHz for ^{13}C{^{1}H}, 162 MHz for ^{31}P{^{1}H}, 376 MHz for ^{19}F{^{1}H} and 111 MHz for ^{129}Xe NMR, and a MAS rate of 10 kHz. For ^{13}C{^{1}H} CP/MAS a sequence with a variable X-amplitude spin-lock pulse[5] and spinal64 proton decoupling was used. Relaxation time for ^{1}H and contact time for ^{31}P{^{1}H} CP/MAS, ^{13}C{^{1}H} CP/MAS, ^{13}C{^{1}H} NQS and fslg-HETCOR[4] NMR experiments were optimized for each

compound. ^{129}Xe NMR experiments were optimized using a solid state sample of XeF_2 and the chemical shifts (δ) referenced to Xe gas in the zero pressure limit of (–5460 ppm) relative to neat liquid $O=XeF_4$ as previously reported.[6] All $^{13}C\{^1H\}$ CP/MAS spectra were referenced to adamantane (upfield methine resonance, δ = 29.5 ppm)[7] on a scale where δ(TMS) = 0 ppm as a secondary reference. The temperature for NMR experiments was externally calibrated using lead nitrate ($PbNO_3$). For the fslg-HETCOR external calibration of against tyrosine at different temperatures was undertaken.[8]

Solid-state NMR samples preparation

SSNMR samples were prepared on either in a MBraun argon-filled glovebox or using a custom-built glassware (Figure S15) for capping a solid-state NMR zirconia rotor under a flush of xenon gas. Crystalline samples (typically 50-75 mg) were packed and capped off into zirconia VT solid-state rotor (4 mm) and transferred to a SSNMR instrument for data acquisition.

Mass spectrometry data

Electrospray ionization mass spectrometry (ESI-MS) was carried out using a Bruker MicrOTOF instrument directly connected to a modified Innovative Technology glovebox.[9] Typical acquisition parameters were used (sample flow rate: 4 μL min^{-1}, nebulizer gas pressure: 0.4 bar, drying gas: argon at 333 K flowing at 4 L min^{-1}, capillary voltage: 4.5 kV, exit voltage: either 15 or 60 V). The spectrometer was calibrated using a mixture of tetra alkyl ammonium bromides $[N(C_nH_{2n+1})_4]Br$ (n = 2-8, 12, 16 and 18) in dichloromethane. Samples were diluted to a concentration of *ca.* < 1 × 10^{-6} M in the appropriate solvent (dichloromethane, 1,2-difluorobenzene or acetone) before sampling by ESI-MS.

SYNTHETHIC PROCEDURES AND CHARACTERIZATION DATA

Solution preparation and characterization of [Rh($Cy_2PCH_2PCy_2$)($\eta^2\eta^2$–NBD)][(CH_2Cl_2)$_{0.75}$⊂BAr$^F{}_4$] [2–NBD][(CH_2Cl_2)$_{0.75}$⊂BAr$^F{}_4$].

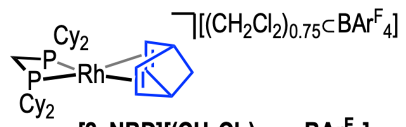

[2–NBD][(CH_2Cl_2)$_{0.75}$⊂BAr$^F{}_4$]

One Young's flask was charged with [Rh($Cy_2PCH_2PCy_2$)($\eta^2\eta^2$–COD)][BAr$^F{}_4$][3] (200 mg, 135 µmol) and dissolved in 1,2-difluorobenzene (5 mL). The orange solution was freeze-pump-thaw degassed three times before H_2 gas (1 bar) was added. The reaction mixture was allowed to stir for one hour resulting in lighter orange solution, and then H_2 and solvent were removed *in vacuo* until dryness. This yielded the compound [Rh($Cy_2PCH_2PCy_2$)(1,2-$F_2C_6H_4$)][BAr$^F{}_4$] as concluded form the ^{1}H and ^{31}P NMR spectroscopic data being identical to that previously published.[3] The resulting pale brown solid was washed with *n*-pentane (3 × 5 mL) and dissolved in dichloromethane (2 mL). Addition of an excess of norbornadiene (0.14 mL, 1.377 mmol) and stirring for one hour resulted in a dark orange solution. The resulting solution was filtered *via* cannula into a Young's crystallization tube. Crystals suitable for an X-ray diffraction study of **[2–NBD][(CH_2Cl_2)$_{0.75}$⊂BAr$^F{}_4$]** were obtained by layering the resulting solution with *n*-pentane. **[2–NBD][(CH_2Cl_2)$_{0.75}$⊂BAr$^F{}_4$]** crystallised as orange prisms. Yield: 180 mg, 116 µmol, 86%.

For SS NMR characterisation, a crystalline sample of freshly prepared **[2–NBD][(CH_2Cl_2)$_{0.75}$⊂BAr$^F{}_4$]** (75 mg, 49 µmol) was packed into a 4 mm zirconia rotor in an argon filled glove box. Then, the zirconia rotor was capped and transferred to SSNMR spectrometer for immediate data acquisition.

Note: The crystalline compound **[2–NBD][(CH_2Cl_2)$_{0.75}$⊂BAr$^F{}_4$]** was dried over a flush of argon for 45 mins and then it was stored in a freezer operating at –34 °C inside an argon filled glove box to prevent CH_2Cl_2. It was observed the loss of CH_2Cl_2 when storing the compound in an atmosphere of argon over prolongated periods of time (> 3 weeks) at room temperature and ambient pressure or when the sample was subjected to dynamic vacuum (< 1 × 10^{-2} mbar).

^{1}H NMR (500 MHz, CD_2Cl_2, 298 K): δ 7.72 (m, 8 H, *ortho*-BAr$^F{}_4$), 7.56 (s, 4 H, *para*-BAr$^F{}_4$), 5.68 (br m, 4 H, alkene-NBD), 5.33 (s, ~ 1.5 H, CH_2Cl_2) 4.20 (s, 2 H, bridgehead-NBD), 3.02 (td, 2 H, $^2J_{PH}$ = 9.4 Hz, $^3J_{PhH}$ = 1.1 Hz, PCH_2P), 1.99-1.74 (m, 23 H, Cy-phosphine), 1.67 (s, 2 H, bridge-CH_2 NBD) and 1.41-1.27 (m, 21 H, Cy-phosphine).

^{1}H NMR (500 MHz, acetone-*d*$_6$, 298 K): δ 7.79 (m, 8 H, *ortho*-BAr$^F{}_4$), 7.67 (s, 4 H, *para*-BAr$^F{}_4$), 5.90 (br m, 4 H, alkene-NBD), 5.61 (s, ~ 1.5 H, CH_2Cl_2) 4.26 (s, 2 H, bridgehead-NBD), 3.46 (td, 2 H, $^2J_{PH}$ = 9.6 Hz, $^3J_{PhH}$ = 1.4 Hz, PCH_2P), 2.15-1.70 (m, 22 H, Cy-phosphine), 1.66 (s, 2 H, bridge-CH_2 NBD) and 1.59-1.28 (m, 22 H, Cy-phosphine).

^{31}P{^{1}H} NMR (162 MHz, CD_2Cl_2, 298 K): δ –22.5 (d, $^1J_{RhP}$ = 133 Hz).

^{31}P{^{1}H} NMR (162 MHz, acetone-*d*$_6$, 298 K): δ –23.1 (d, $^1J_{RhP}$ = 132 Hz).

^{13}C{^{1}H} NMR (125.8 MHz, acetone-*d*$_6$, 298 K): δ 162.6 (q, $^1J_{CB}$ = 49.8 Hz, *ipso*-BAr$^F{}_4$), 135.5 (s, *ortho*-BAr$^F{}_4$), 129.9 (qq, $^3J_{CB}$ = 3.0 Hz, $^2J_{CF}$ = 31.5 Hz, *meta*-BAr$^F{}_4$), 125.3 (q, $^1J_{CF}$ = 271.8 Hz, *CF_3*-BAr$^F{}_4$), 118.4 (sept, $^3J_{CF}$ = 3.7 Hz, *para*-BAr$^F{}_4$), 90.3 (m, alkene-NBD); 72.1 (bridge-CH_2 NBD); 57.3 (bridgehead-CH NBD); 54.9 (CH_2Cl_2); 36.1(t, $^3J_{CP}$ = 9.2 Hz, phosphine); 30-29.3 (phosphine resonances overlapped with acetone-*d*$_6$), 27.4 (m, phosphine), 26.4 (s, phosphine).

^{31}P{^{1}H} SSNMR (162 MHz, 10 kHz spin rate, 294 K): δ –24.6 (br d, *J*(Rh-P) ~ 120 Hz), –28.0 (br s, not resolved resonance).

^{13}C{^{1}H} SSNMR (101 MHz, 10 kHz spin rate, 294 K): δ 163.8 (br m, $\nu_{1/2}$ = 200 Hz overlapping C_{ipso} resonances, BAr$^F{}_4$); 135.3, 134.5 and 133.5 (*ortho*-BAr$^F{}_4$ resonances); 130.7 (*meta*-BAr$^F{}_4$); 124.9 (v br, $\nu_{1/2}$ = 275 Hz, overlapping CF_3 resonances, BAr$^F{}_4$); 118.0 and 116.9 (both *para*-BAr$^F{}_4$ resonances); 94.7, 91.7, 87.7 and 87.1 (alkene-NBD resonances); 69.6 (bridge-CH_2 NBD); 56.3 and 56.0 (both bridgehead-

CH resonances NBD); 52.0 (CH_2Cl_2); 40.2 (br); 34.8, 34.0, 32.4, 30.2, 28.0 and 26.0 (multiple overlapping aliphatic resonances, phosphine).

$^1H/^{13}C\{^1H\}$ projections from Frequency Switched Lee-Goldburg HETCOR SSNMR (^1H, 400 MHz, 10 kHz spin rate, 294K): δ, BAr$^F_4$: 7.60/135.3, 7.60/134.5, 133.5/7.60, 130.7/7.60, 7.10/118.0, and 7.10/116.9; alkene-NBD: 5.05/94.7, 5.05/91.7, 5.10/87.7 and 5.10/87.1; bridge-CH_2 NBD: –1.80/69.6; bridgehead-CH NBD: 2.80/56.3 and 3.10/56.0; CH_2Cl_2: 3.05/52.0; aliphatic phosphine: 1.20/34.8, 1.00/34.8, 1.00/34.0, 1.05/32.4, 0.95/30.2, 1.00/28.0 and 1.00/26.0

Elemental analysis found (calculated) for $C_{64.75}H_{67.5}BCl_{1.5}F_{24}P_2Rh$: C, 50.81 (50.52); H, 4.55 (4.53)%.

HRMS (ESI) m/z found (calculated) for $C_{32}H_{54}P_2Rh$ [M]$^+$: 603.2773 (603.2750).

NMR spectra of [2–NBD][(CH_2Cl_2)$_{0.75}$⊂BAr$^F_4$].

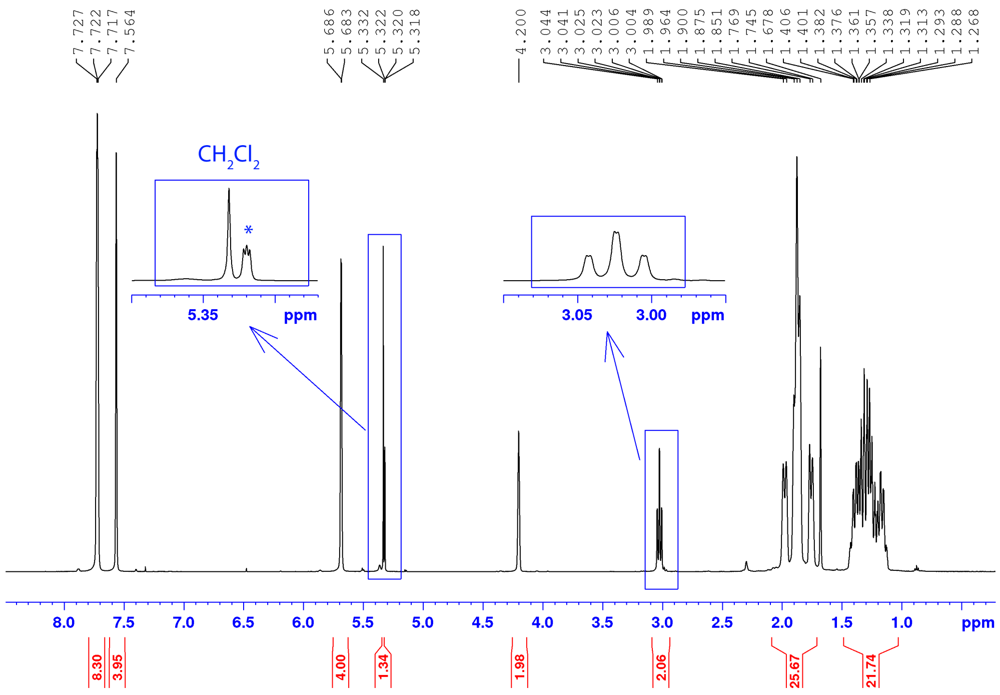

Figure S1. ^1H NMR (500 MHz, CD_2Cl_2, 298 K) spectrum of **[2–NBD][(CH_2Cl_2)$_{0.75}$⊂BAr$^F_4$]**. CD_2Cl_2 residual solvent peak marked with a blue asterisk.

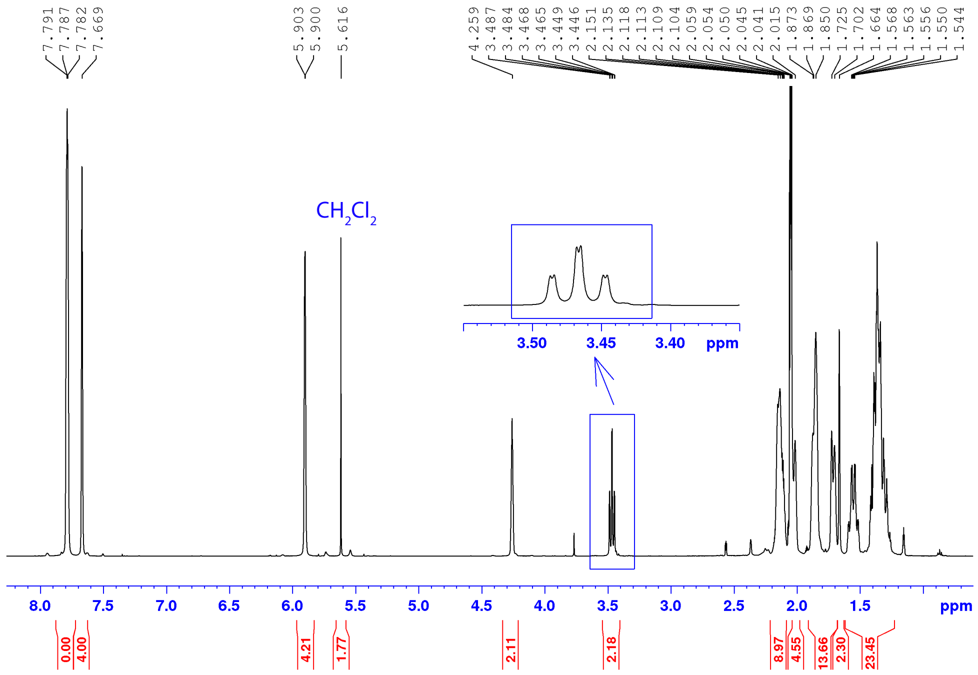

Figure S2. ^{1}H NMR (500 MHz, acetone-d_6, 298 K) spectrum of **[2–NBD][$(CH_2Cl_2)_{0.75}$⊂BAr$^F_4$]**.

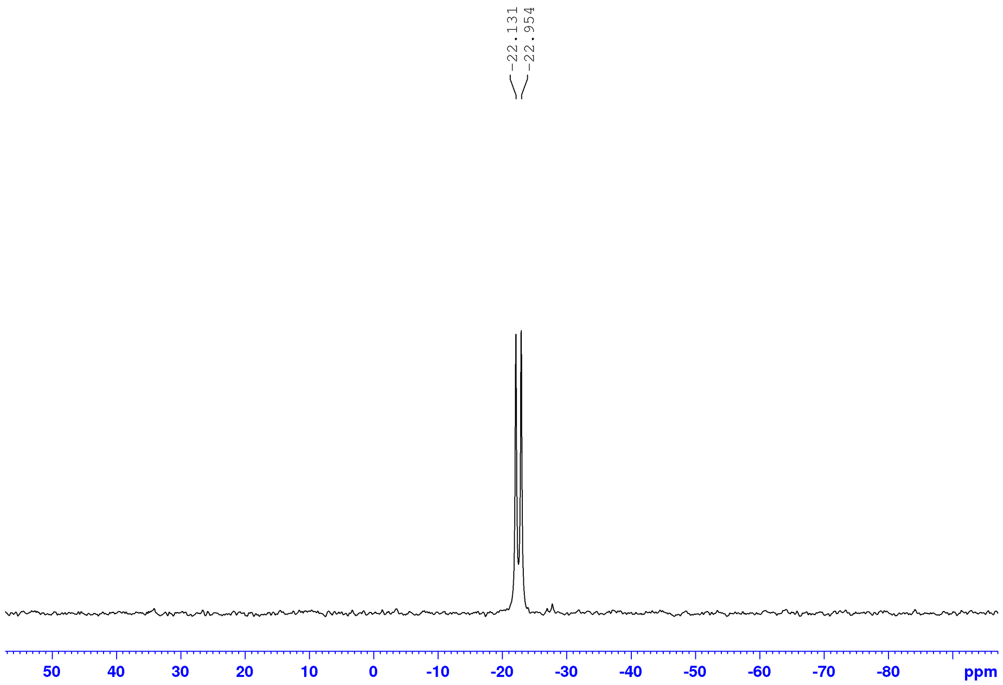

Figure S3. $^{31}P\{^{1}H\}$ NMR (162 MHz, CD_2Cl_2, 298 K) spectrum of **[2–NBD][$(CH_2Cl_2)_{0.75}$⊂BAr$^F_4$].**

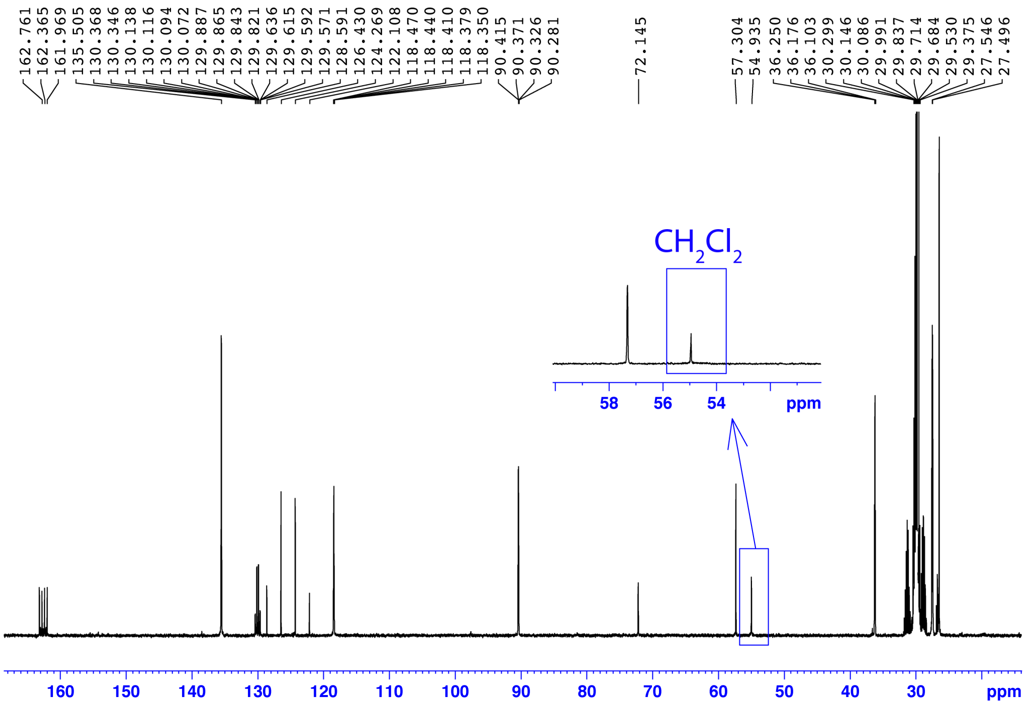

Figure S4. $^{13}C\{^1H\}$ NMR (158.5 MHz, acetone-d_6, 298 K) spectrum of **[2–NBD][(CH_2Cl_2)$_{0.75}$⊂BAr$^F{}_4$].**

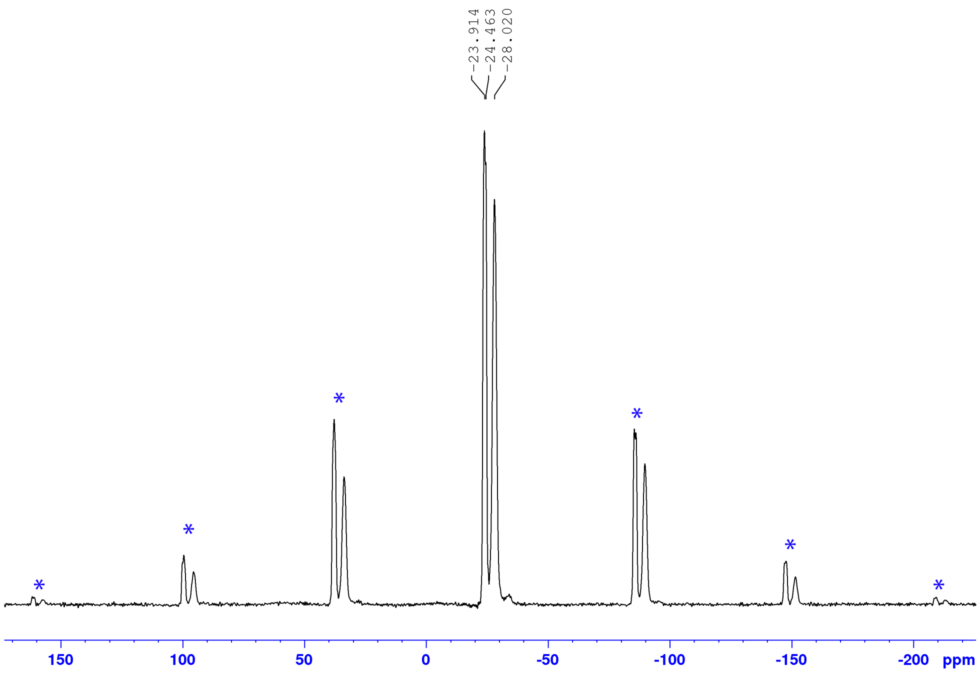

Figure S5. $^{31}P\{^1H\}$ SSNMR (162 MHz, 10 kHz spin rate, 294 K) spectrum of **[2–NBD][(CH_2Cl_2)$_{0.75}$⊂BAr$^F{}_4$]**. Spinning sidebands are marked with blue asterisks.

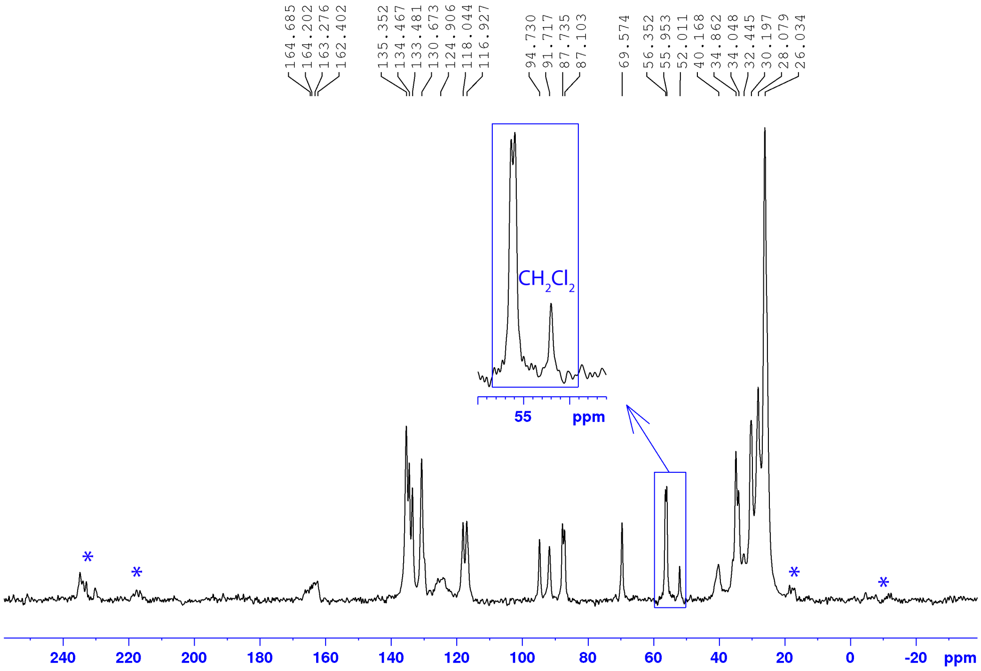

Figure S6: $^{13}C\{^1H\}$ SSNMR (101 MHz, 10 kHz spin rate, 294 K) spectrum of **[2–NBD][(CH$_2$Cl$_2$)$_{0.75}$⊂BAr$^F_4$]**. Spinning sidebands are marked with blue asterisks.

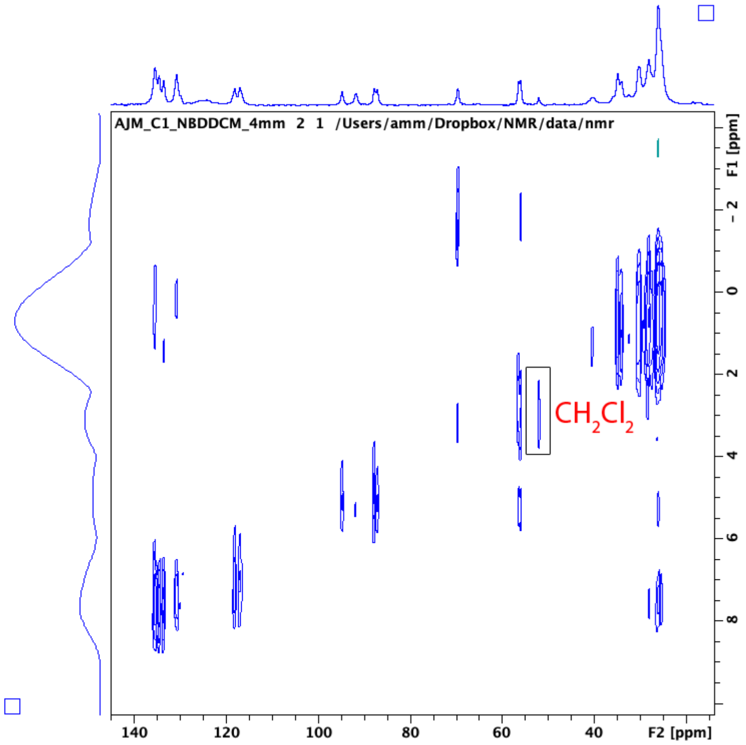

Figure S7. $^1H/^{13}C$ Frequency Switched Lee-Goldburg HETCOR SSNMR spectrum of **[2–NBD][(CH$_2$Cl$_2$)$_{0.75}$⊂BAr$^F_4$]** at 294 K.

Solid-state preparation and characterization of [Rh($Cy_2PCH_2PCy_2$)($\eta^2\eta^2$–NBD)][BAr^F_4] [2–NBD][BAr^F_4].

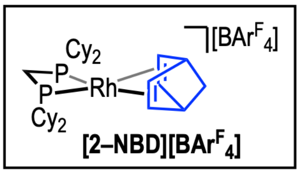

One Young's flask was charged with single crystals (orange prisms) of **[2–NBD][(CH_2Cl_2)$_{0.75}$⊂BAr^F_4]** (120 mg, 78 μmol) in an argon filled glove box. Then, the crystalline compound was subjected to dynamic vacuum (< 1 × 10^{-2} mbar) for 24 hours at room temperature to give [Rh($Cy_2PCH_2PCy_2$)($\eta^2\eta^2$–NBD)][BAr^F_4] **[2–NBD][BAr^F_4]** in almost quantitative yield. Little colour change was observed, though the colour of the crystals turned slightly lighter progressively. After this period of time, the sample was backfilled with argon (0.5 bar, 298 K). Then, it was stored into an argon filled glove box. This transformation gave suitable single crystals of **[2–NBD][BAr^F_4]** for an X-ray diffraction study. Yield: 110 mg, 75 μmol, 96%.

For SS NMR characterisation, a crystalline sample of **[2–NBD][BAr^F_4]** (75 mg, 51 μmol) was packed into a 4 mm zirconia rotor in an argon filled glove box. Then, the zirconia rotor was capped and transferred to SSNMR spectrometer for immediate data acquisition.

^{1}H NMR (400 MHz, CD_2Cl_2, 298 K): δ 7.72 (m, 8 H, *ortho*-BAr^F_4), 7.56 (s, 4 H, *para*-BAr^F_4), 5.69 (br s, 4 H, alkene-NBD), 4.21 (s, 2 H, bridgehead-NBD), 3.02 (t, 2 H, $^2J_{PH}$ = 9.3 Hz), 2.00-1.76 (m, 23 H, Cy-phosphine), 1.69 (s, 2 H, bridge-CH_2 NBD), 1.41-1.16 (m, 21 H, Cy-phosphine).

^{1}H NMR (400 MHz, acetone-d_6, 298 K): δ 7.79 (m, 8 H, *ortho*-BAr^F_4), 7.67 (s, 4 H, *para*-BAr^F_4), 5.90 (br m, 4 H, alkene-NBD), 4.26 (s, 2 H, bridgehead-NBD), 3.46 (td, 2 H, $^2J_{PH}$ = 9.3 Hz), 2.13-1.70 (m, 22 H, Cy-phosphine), 1.66 (s, 2 H, bridge-CH_2 NBD) and 1.56-1.28 (m, 22 H, Cy-phosphine).

^{31}P{^{1}H} NMR (162 MHz, CD_2Cl_2, 298 K): δ –22.5 (d, $^1J_{RhP}$ = 134 Hz).

^{31}P{^{1}H} NMR (162 MHz, acetone-d_6, 298 K): δ –23.0 (d, $^1J_{RhP}$ = 132 Hz).

^{13}C{^{1}H} NMR (125.8 MHz, acetone-d_6, 298 K): δ 162.5 (q, $^1J_{CB}$ = 49.8 Hz, *ipso*-BAr^F_4), 135.5 (s, *ortho*-BAr^F_4), 129.9 (qq, $^3J_{CB}$ = 3.0 Hz, $^2J_{CF}$ = 31.2 Hz, *meta*-BAr^F_4), 125.3 (q, $^1J_{CF}$ = 271.8 Hz, *CF_3*-BAr^F_4), 118.4 (sept, $^3J_{CF}$ = 3.7 Hz, *para*-BAr^F_4), 90.3 (m, alkene-NBD); 72.1 (bridge-CH_2 NBD); 57.3 (bridgehead-CH NBD); 36.1(t, $^3J_{CP}$ = 9.0 Hz, phosphine); 30-29.3 (phosphine resonances overlapped with acetone-d_6), 27.4 (m, phosphine), 26.4 (s, phosphine).

^{31}P{^{1}H} SSNMR (162 MHz, 10 kHz spin rate, 294 K): δ –23.3 (br dd, J_{RhP} ~ 120 Hz, J_{PP} ~ 70 Hz), –27.1 (br dd, J_{RhP} ~ 115 Hz, J_{PP} ~ 65 Hz)).

^{13}C{^{1}H} SSNMR (101 MHz, 10 kHz spin rate, 294 K): δ 164.0 (br m, $\nu_{1/2}$ = 275 Hz overlapping C_{ipso} resonances, BAr^F_4); 134.7 and 133.6 (both *ortho*-BAr^F_4); 130.4 and 129.7 (both *meta*-BAr^F_4); 124.7 (v br, $\nu_{1/2}$ = 375 Hz, overlapping CF_3 resonances, BAr^F_4); 118.1, 117.2, and 116.0 (*para*-BAr^F_4 resonances); 94.5, 92.3 and 87.7 (alkene-NBD resonances); 69.6 (bridge-CH_2 NBD); 56.4 and 55.9 (both bridgehead-CH resonances NBD); 39.1, 35.5, 34.9, 33.5, 31.7, 30.3, 29.8, 27.9, 27.3 and 25.8 (multiple overlapping aliphatic resonances, phosphine).

Elemental analysis found (calculated) for $C_{64}H_{66}BF_{24}P_2Rh$: C, 52.32 (52.40); H, 4.65 (4.54)%.

HRMS (ESI) m/z found (calculated) for $C_{32}H_{54}P_2Rh$ [M]$^+$: 603.2761 (603.2750).

NMR spectra of [2–NBD][BAr$^F{}_4$].

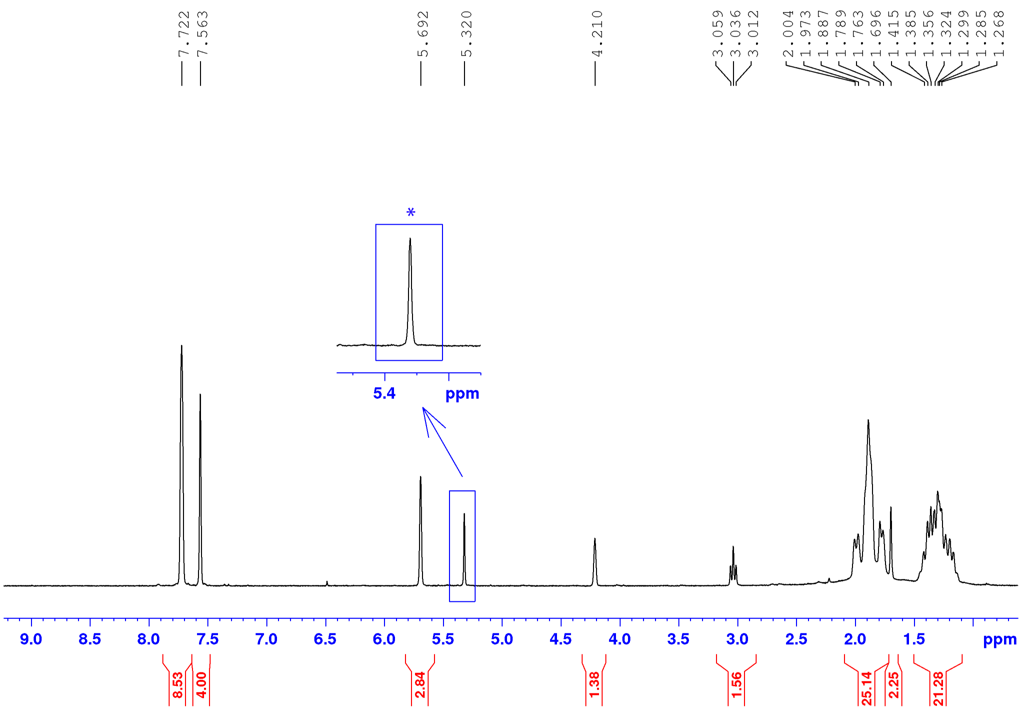

Figure S8. ^{1}H NMR (400 MHz, CD_2Cl_2, 298 K) spectrum of **[2–NBD][BAr$^F{}_4$]**. CD_2Cl_2 residual solvent peak marked with a blue asterisk. CH_2Cl_2 resonance is absent.

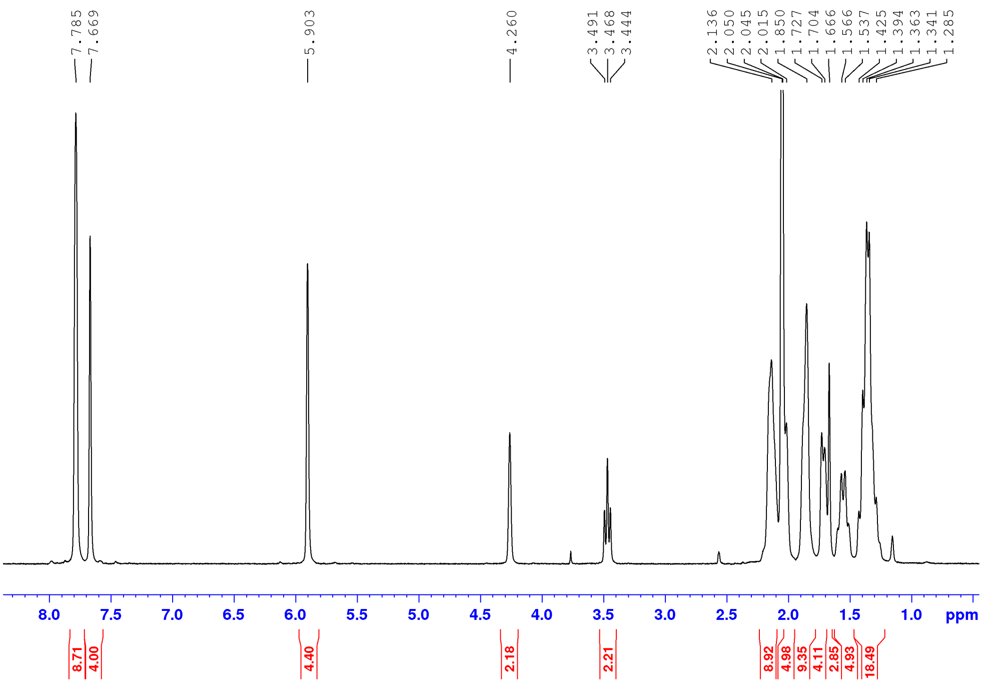

Figure S9. ^{1}H NMR (400 MHz, acetone-d_6, 298 K) spectrum of **[2–NBD][BAr$^F{}_4$]**. CH_2Cl_2 resonance is absent.

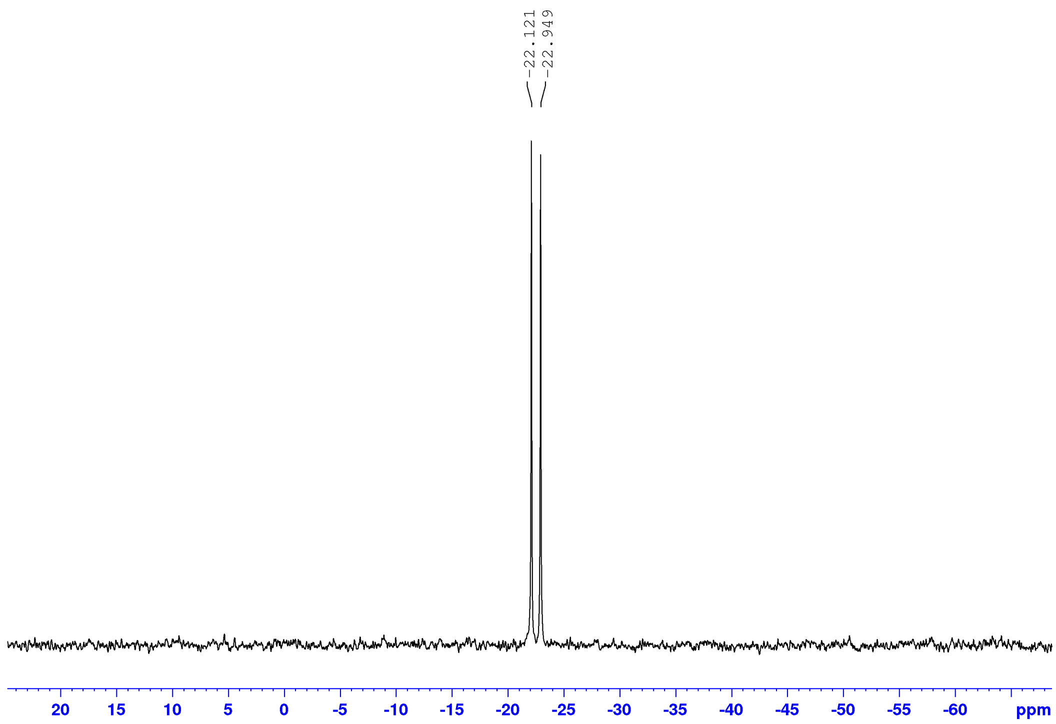

Figure S10. $^{31}P\{^{1}H\}$ NMR (162 MHz, CD_2Cl_2, 298 K) spectrum of **[2–NBD][BAr$^{F}_{4}$].**

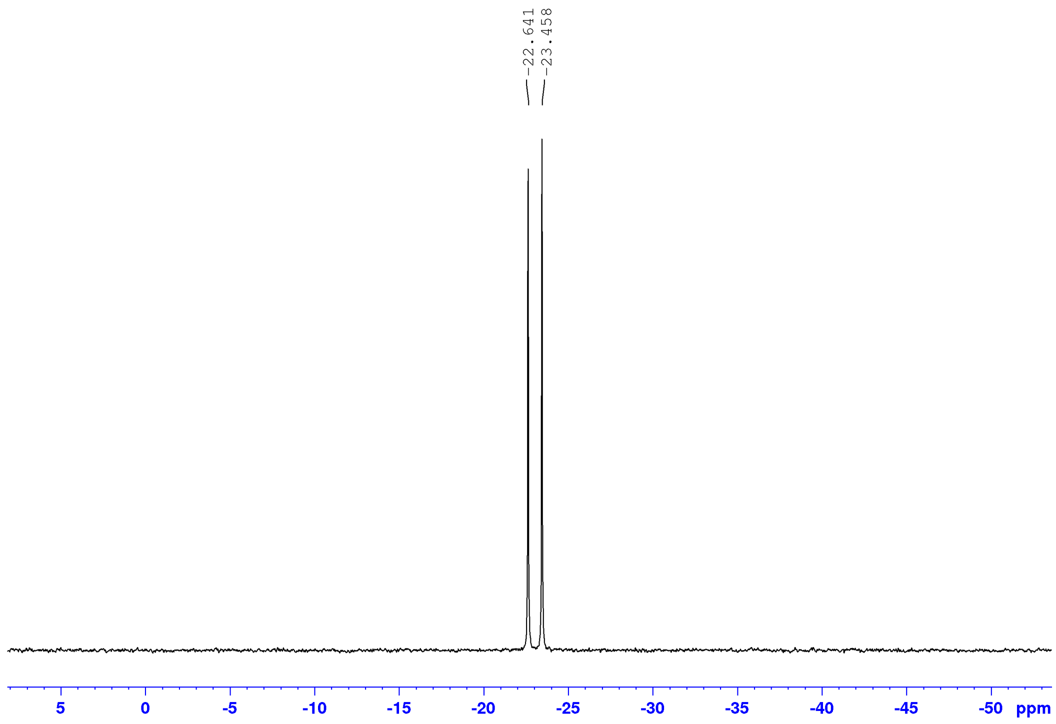

Figure S11. $^{31}P\{^{1}H\}$ NMR (162 MHz, acetone-d_6, 298 K) spectrum of **[2–NBD][BAr$^{F}_{4}$].**

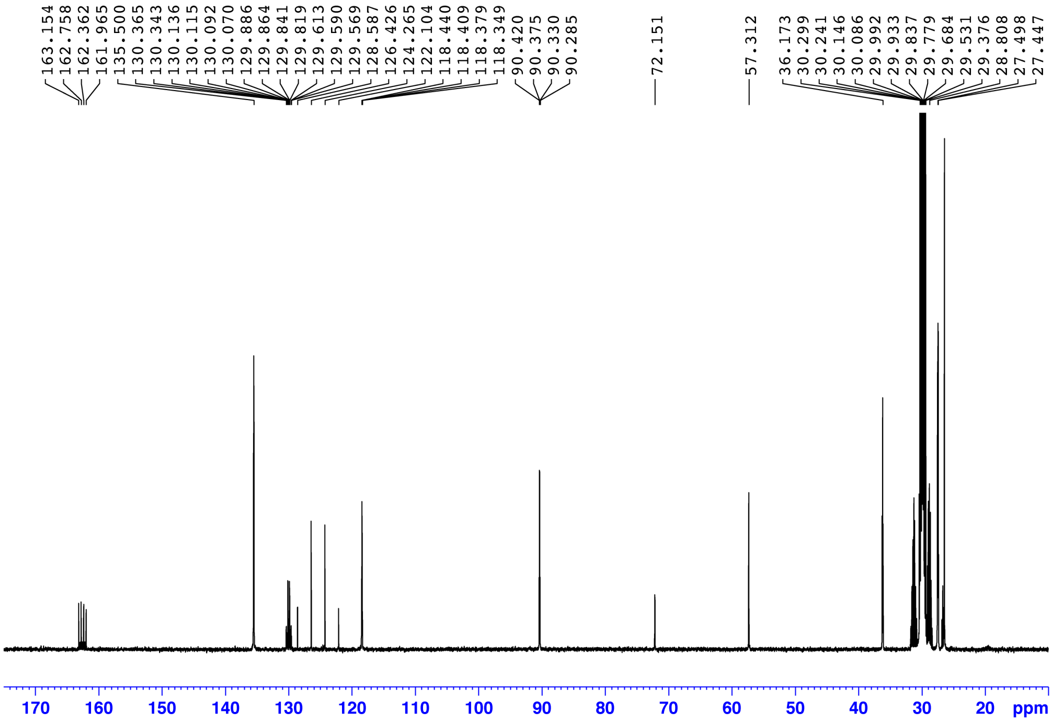

Figure S12. $^{13}C\{^{1}H\}$ NMR (158.5 MHz, acetone-d_6, 298 K) spectrum of **[2–NBD][BAr$^F_4$].** CH_2Cl_2 resonance is absent.

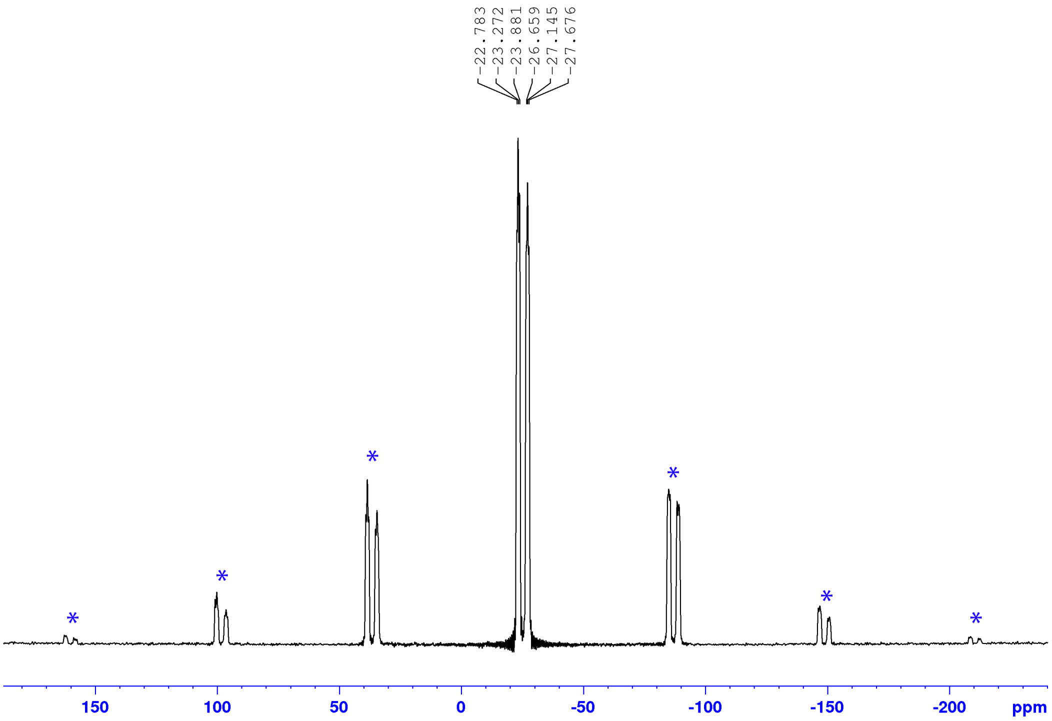

Figure S13. $^{31}P\{^{1}H\}$ SSNMR (162 MHz, 10 kHz spin rate, 294 K) spectrum of **[2–NBD][BAr$^F_4$]**. Spinning sidebands are marked with blue asterisks.

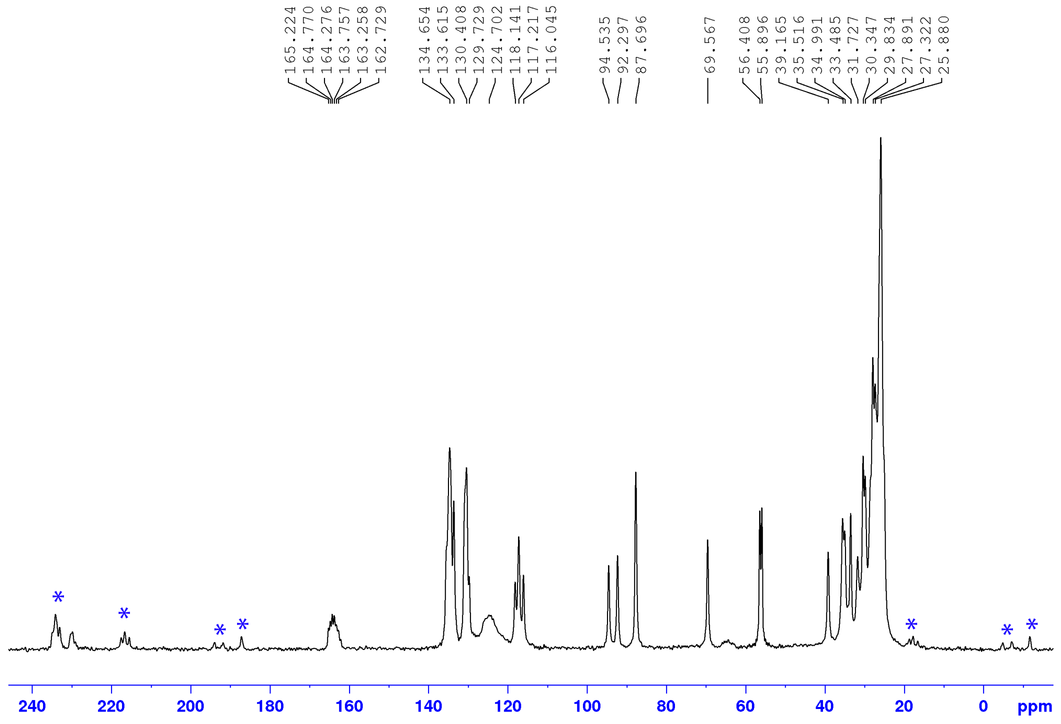

Figure S14: $^{13}C\{^{1}H\}$ SSNMR (101 MHz, 10 kHz spin rate, 294 K) spectrum of **[2–NBD][BAr$^F_4$]**. Spinning sidebands are marked with blue asterisks. CH_2Cl_2 resonance at δ 52.0 is absent.

Solid-state transformation of [2–NBD][BAr$^F_4$] into [2–NBD][$(CH_2Cl_2)_{0.75}$⊂BAr$^F_4$].

One Young's flask was charged with single crystals (light orange prisms) of freshly prepared [Rh($Cy_2PCH_2PCy_2$)($\eta^2\eta^2$–NBD)][BAr$^F_4$] **[2–NBD][BAr$^F_4$]** (50 mg, 34 µmol) in an argon filled glove box. Then, the crystalline compound was subjected to dynamic vacuum (< 1 × 10^{-2} mbar) for 1 min at room temperature. Then, the headspace of Young's flask containing the crystalline material was backfilled with vapours of CH_2Cl_2 by connecting it to a second Young's flask containing CH_2Cl_2 (5 mL). This operation was repeated after 12 hours (x 4 times) to give **[2–NBD][BAr$^F_4$]** in almost quantitative yield after 48 hours. After this period of time, the sample was flushed with argon gas at room temperature (1 min). Little colour change was observed, though the colour of the crystals turned slightly darker progressively. Then, it was stored into an argon filled glove box. This transformation gave suitable single crystals of [Rh($Cy_2PCH_2PCy_2$)($\eta^2\eta^2$–NBD)][$(CH_2Cl_2)_{0.75}$⊂BAr$^F_4$] **[2–NBD][$(CH_2Cl_2)_{0.75}$⊂BAr$^F_4$]** for an X-ray diffraction study. Yield: 47 mg, 31 µmol, 91%.

Characterisation data, including solution NMR and $^{31}P\{^{1}H\}$ SSNMR spectroscopic data and single-crystal X-ray diffraction studies, for **[2–NBD][$(CH_2Cl_2)_{0.75}$⊂BAr$^F_4$]**, prepared by solid-state route from **[2–NBD][BAr$^F_4$]** is identical to that of the compound prepared by solution methods from [Rh($Cy_2PCH_2PCy_2$)($\eta^2\eta^2$–COD)][BAr$^F_4$].[3]

Solid state preparation and characterization of [Rh($Cy_2PCH_2PCy_2$)($\eta^2\eta^2$–NBD)][$(Xe)_{0.5}\subset BAr^F_4$] [2–NBD][$(Xe)_{0.5}\subset BAr^F_4$] from [2–NBD][$BAr^F_4$].

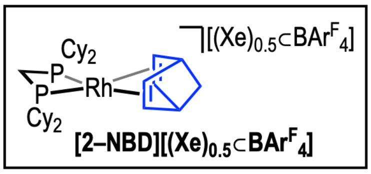

A small batch of crystals of **[2–NBD][BAr^F_4]** (~ 6 mg, ~ 4 μmol) were evacuated (< 1 × 10^{-2} mbar, 1 min) in a medium wall NMR tube (~ 2 mL) fitted with a controlled atmosphere TFE valve. Then, xenon gas (3 bar, 1 week, 298 K) was added. Little colour change was observed, though the colour of the crystals turned slightly lighter progressively. After this time, a suitable crystal (light orange prism) was quickly transferred (< 2 mins) to a pre-cooled diffractometer (150 K) allowing for the study of the molecular structure of the compound being formed. Reaction with xenon under these conditions resulted with the formation of the compound [Rh($Cy_2PCH_2PCy_2$)($\eta^2\eta^2$–NBD)][$(Xe)_{0.5}\subset BAr^F_4$] **[2–NBD][$(Xe)_{0.5}\subset BAr^F_4$]** (see Crystallographic Section for full details).

For SS NMR characterisation, a crystalline sample of **[2–NBD][BAr^F_4]** (75 mg, 34 μmol) was packed into a 4 mm zirconia rotor in an argon filled glove box. Then, the zirconia rotor was placed inside a custom-built Young's glass adaptor (see Figure S15) and the sample was evacuated (< 1 × 10^{-2} mbar, 1 min) and backfilled with xenon gas (3 bar, 1 week, 298 K). After this time, zirconia rotor was capped using the Young's glass adaptor under a flow of xenon and then it was transferred to SSNMR spectrometer for immediate data acquisition. Note that the SSNMR characterization of compound **[2–NBD][$(Xe)_{0.5}\subset BAr^F_4$]** was undertaken with the compound capped in the zirconia rotor under an atmosphere of xenon gas.

Attempts to obtain meaningful elemental analyses failed due to the rapid loss of xenon from **[2–NBD][$(Xe)_{0.5}\subset BAr^F_4$]** to give **[2–NBD][$BAr^F_4$]** consistently.

^{129}Xe SSNMR (111 MHz, 10 kHz spin rate, 294 K): δ –5275 and –5460 (xenon gas).

Note: **^{129}Xe EXSY SSNMR** experiments with diverse mixing times (1.2 sec – 5 msec) were collected, no exchange peaks were observed between the resonance for the compound **[2–NBD][$(Xe)_{0.5}\subset BAr^F_4$]** (–5275 ppm) and that of the free xenon gas (–5275 ppm) at room temperature.

^{31}P{^{1}H} SSNMR (162 MHz, 10 kHz spin rate, 294 K): δ –23.8 and –27.2 (both br).

^{19}F{^{1}H} SSNMR (376 MHz, 10 kHz spin rate, 294 K): δ –63.2 (br).

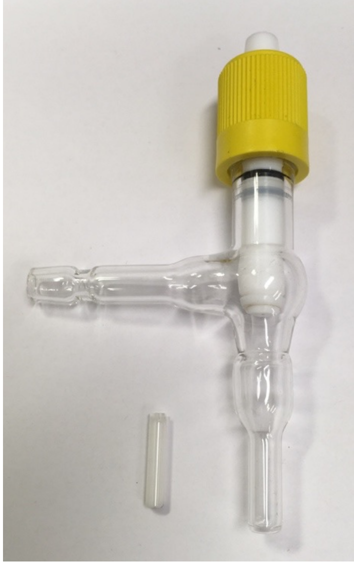

Figure S15. Custom built glassware (right) for capping a solid-state NMR zirconia rotor (left) under a flush of xenon gas.

Solid-state transformation of [2–NBD][$(Xe)_{0.5}\subset BAr^F{}_4$] into [2–NBD][$BAr^F{}_4$].

One zirconia rotor was charged with single crystals (orange prisms) of freshly prepared [Rh($Cy_2PCH_2PCy_2$)($\eta^2\eta^2$–NBD)][$BAr^F{}_4$] **[2–NBD][$BAr^F{}_4$]** (75 mg, 34 μmol) in an argon filled glove box. Then, the zirconia rotor was placed inside a custom-built Young's glass adaptor (Figure S15) and the sample was evacuated (< 1 × 10^{-2} mbar, 1 min) and backfilled with xenon gas (3 bar, 1 week, 298 K). After this time, zirconia rotor was capped using the Young's glass adaptor under a flow of xenon and then it was transferred to SSNMR spectrometer for immediate data acquisition. Spectroscopic SSNMR data proved that the formation of **[2–NBD][$(Xe)_{0.5}\subset BAr^F{}_4$]** under an atmosphere of xenon gas (ambient pressure). Then, the zirconia rotor containing **[2–NBD][$(Xe)_{0.5}\subset BAr^F{}_4$]** under an atmosphere of xenon gas was transferred back to a custom-built Young's glass adaptor (see Figure S15) and the zirconia rotor was uncapped. The sample was flushed with argon gas for 2 mins. After this time, the zirconia rotor was capped under a flush of argon using the custom-built Young's glass adaptor and transferred back to the SSNMR spectrometer for immediate data acquisition. Spectroscopic data proved the rapid loss of xenon and formation of **[2–NBD][$BAr^F{}_4$]** in almost quantitative yield.

For single crystal X-ray studies, two batches of crystals of **[2–NBD][$BAr^F{}_4$]** (~ 6 mg, ~ 4 μmol, each) were evacuated (< 1 × 10^{-2} mbar, 1 min) in two medium wall NMR tube (~ 2 mL, each) fitted with a controlled atmosphere TFE valve. Then, xenon gas (3 bar, 1 week, 298 K) was added to both. After this time, one of the NMR tubes was open and a suitable crystal (light orange prism) was quickly transferred (< 2 mins) to a pre-cooled diffractometer (150 K) allowing for the study of the molecular structure of the compound being formed. This proved that **[2–NBD][$(Xe)_{0.5}\subset BAr^F{}_4$]** was formed. Then, the second NMR tube was open under a flush of argon gas and flushed for 2 mins. After this time, a suitable crystal (light orange prism) was quickly transferred (< 2 mins) to a pre-cooled diffractometer (150 K) allowing for the study of the molecular structure of the compound being formed. This proved that **1** was formed as a result of the loss of xenon under these conditions.

SSNMR spectroscopic characterization and single crystal X-ray diffraction data of compounds **[2–NBD][$BAr^F{}_4$]** and **[2–NBD][$(Xe)_{0.5}\subset BAr^F{}_4$]** obtained during these experiments are essentially identical to that of the freshly prepared compounds following the synthetic protocols described here.

Solid state temporal studies of [2–NBD][$BAr^F{}_4$] xenon gas

The solid/gas reaction of **[2–NBD][$BAr^F{}_4$]** with xenon gas was monitored by single crystal X-ray diffraction studies by evacuating (< 1 × 10^{-2} mbar, 1 min, each) small batches of crystals of **[2–NBD][$BAr^F{}_4$]** (~ 6 mg, ~ 4 μmol, each) in medium wall NMR tubes (~ 2 mL, each) fitted with a controlled atmosphere TFE valves and then allowing them to react with xenon gas (3 bar, 298 K) for various periods of time (1 day, 1 week and 3 weeks). After each time, a suitable crystal (light orange prism) was quickly transferred (< 2 mins) to a pre-cooled diffractometer (150 K) allowing for the study of the molecular structure of the compound being formed. Reaction with xenon under these conditions resulted with the formation of the compound [Rh($Cy_2PCH_2PCy_2$)($\eta^2\eta^2$–NBD)][$(Xe)_{0.5}\subset BAr^F{}_4$] **[2–NBD][$(Xe)_{0.5}\subset BAr^F{}_4$]** (see Crystallographic Section for full details) after 1 or 3 weeks. However, no reaction was observed after 1 day and full recovery of **[2–NBD][$BAr^F{}_4$]** was obtained, as proved by single crystal X-ray diffraction studies of the molecular structure of **[2–NBD][$BAr^F{}_4$]**, and no evidence of xenon in the crystal lattice.

Solid state recharging studies of [2–NBD][$BAr^F{}_4$] xenon gas ([2–NBD][$BAr^F{}_4$] ⇄ [2–NBD][$(Xe)_{0.5}\subset BAr^F{}_4$])

A crystalline sample of **[2–NBD][$BAr^F{}_4$]** (75 mg, 34 μmol) was packed into a 4 mm zirconia rotor in an argon filled glove box. Then, the zirconia rotor was placed inside a custom-built Young's glass adaptor (see Figure S15) and the sample was evacuated (< 1 × 10^{-2} mbar, 1 min) and backfilled with xenon gas

(3 bar, 1 week, 298 K). After this time, zirconia rotor was capped using the Young's glass adaptor under a flow of xenon and then it was transferred to SSNMR spectrometer for immediate data acquisition. The SSNMR data collected matched that of compound **[2–NBD][$(Xe)_{0.5}$⊂$BAr^F{}_4$]**. Then, the zirconia rotor was uncapped under a flush of argon gas using the custom-built Young's glass adaptor and the sample was flushed with argon gas for 2 mins. After this time, the zirconia rotor was capped under a flow of argon and then it was transferred to SSNMR spectrometer for immediate data acquisition. The SSNMR data collected matched that of compound **1** with no evidences of xenon present in the sample. After this, the zirconia rotor was placed back inside the custom-built Young's glass adaptor and the sample was evacuated again (< 1×10^{-2} mbar, 1 min) and backfilled with xenon gas (3 bar, 1 week, 298 K). After this time, zirconia rotor was capped using the Young's glass adaptor under a flow of xenon and then it was transferred to SSNMR spectrometer for immediate data acquisition. The SSNMR data collected matched that of compound **[2–NBD][$(Xe)_{0.5}$⊂$BAr^F{}_4$]**.

NMR spectra of [2–NBD][$(Xe)_{0.5}$⊂$BAr^F{}_4$].

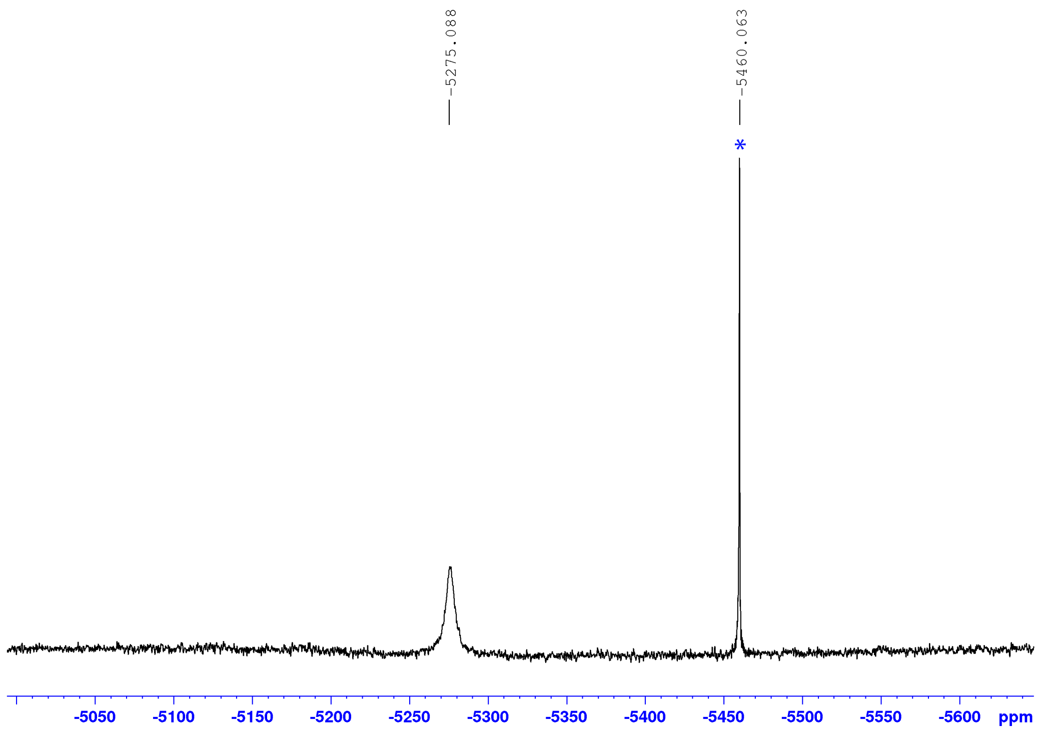

Figure S16. ^{129}Xe SSNMR (111 MHz, 10 kHz spin rate, 294 K) spectrum of **[2–NBD][$(Xe)_{0.5}$⊂$BAr^F{}_4$]**. Residual resonance for xenon gas used in preparing and packing the sample in the zirconia rotor is marked with a blue asterisk.

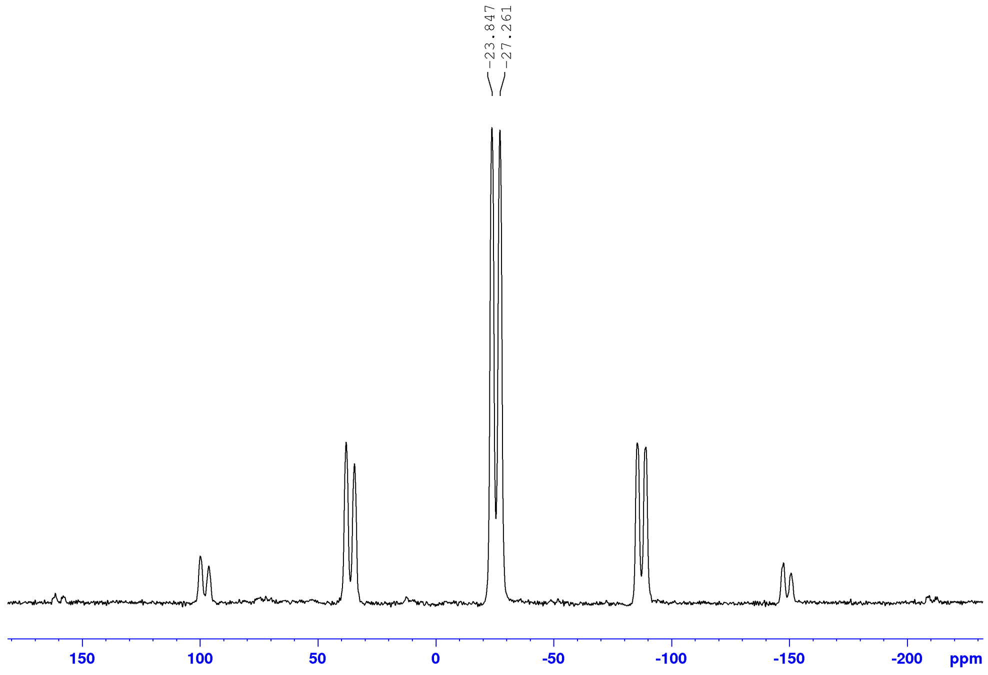

Figure S17. $^{31}P\{^{1}H\}$ SSNMR (162 MHz, 10 kHz spin rate, 294 K) spectrum of **[2–NBD][(Xe)$_{0.5}$⊂BAr$^{F}_{4}$]**. Spinning sidebands are marked with blue asterisks.

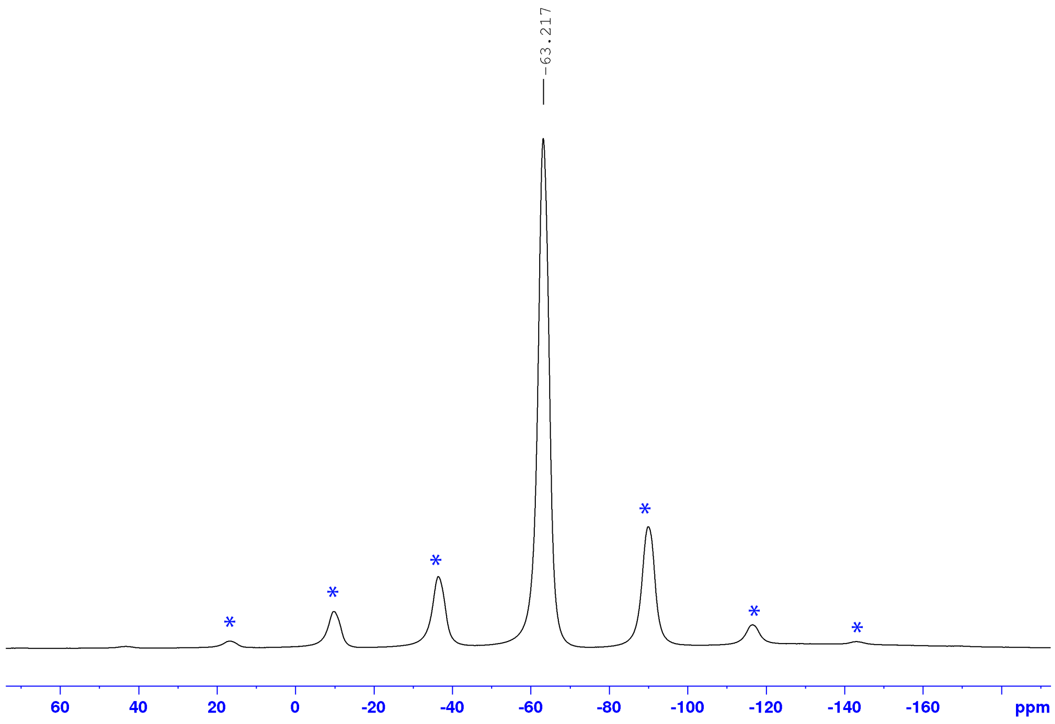

Figure S18. ^{19}F SSNMR (376 MHz, 10 kHz spin rate, 294 K) spectrum of **[2–NBD][(Xe)$_{0.5}$⊂BAr$^{F}_{4}$]**. Spinning sidebands are marked with blue asterisks.

X-RAY CRYSTALLOGRAPHY

Crystal structure determinations

Single-crystal X-ray diffraction data for all the compounds were collected (ω-scans) on Oxford Diffraction/Agilent SuperNova diffractometers with Cu-*Kα* (λ = 1.54184 Å) radiation equipped with nitrogen gas Oxford Cryosystems Cryostream unit[10] at the Oxford Chemical Crystallography Service from the University of Oxford. Diffraction images raw frame data were ultimately reduced using CrysAlisPro.[11] The structures were solved using SHELXT[12] and refined to convergence on F^2 and against all independent reflections by full-matrix least-squares using SHELXL[13] (version 2018/3) in combination with the GUI OLEX2[14] program. All non-hydrogen atoms were refined anisotropically and hydrogen atoms were geometrically placed unless otherwise stated (see specific details for each molecular structure in the text) and allowed to ride on their parent atoms. In several structures some of the CF_3 groups on the $BAr^F{}_4{}^-$ anion were disordered and modelled over two or three main domains, and restrained to maintain sensible geometries. Distances and angles were calculated using the full covariance matrix. Selected crystallographic data are summarized in the text and full details are given in the supplementary deposited CIF files (CCDC 1946574-76). These data can be obtained free of charge from the Cambridge Crystallographic Data Centre via http://www.ccdc.cam.ac.uk/data_request/cif.

Selected crystallographic and refinement data

X-ray crystal structure of [2–NBD][$(CH_2Cl_2)_{0.75}$⊂$BAr^F{}_4$] (CCDC 1946574)

Crystal data for **[2–NBD][$(CH_2Cl_2)_{0.75}$⊂$BAr^F{}_4$]**: $C_{64.75}H_{67.50}BCl_{1.5}F_{24}P_2Rh$, M = 1530.52 g/mol, monoclinic, $P2_1/c$, a = 13.72630(10), b = 18.8374(2), c = 26.5402(3) Å, β = 92.6420(10) °, V = 6855.15 Å^3, Z = 4, λ(Cu-Kα) = 1.54184 Å, T = 150(1) K, orange prism, ρ(calcd, g cm^{-3}) = 1.483, μ (mm^{-1}) = 3.926, 30645 reflections collected, 13750 independent measured reflections (R_{int} = 0.0373), F^2 refinement, R_1(obs, $I > 2\sigma(I)$) = 0.0444, wR_2(all data) = 0.1211, 11381 independent observed reflections [$|F_o| > 4\sigma(|F_o|)$, $2\theta_{max}$ = 153.6°], 768 restrains, 1006 parameters, GOF = 1.033 and residual electron density (e Å^{-3}) = 0.711/-0.964.

Additional details for **[2–NBD][$(CH_2Cl_2)_{0.75}$⊂$BAr^F{}_4$]**: This compound crystallized in the monoclinic space group $P2_1/c$ with one independent molecule in the asymmetric unit. Five CF_3 groups were modelled as disordered. One disordered molecule of dichloromethane CH_2Cl_2 was found in the crystal lattice which was modelled over two main domains. The chemical occupancies for both disordered components freely refined to ~ 0.74 in total (~ 0.65:0.10). Thus, the chemical occupancy for the disordered was set to refine to 0.75 (major:minor components freely refined to 0.651:0.099). When compound **[2–NBD][$(CH_2Cl_2)_{0.75}$⊂$BAr^F{}_4$]** was prepared following the synthetic protocols reported in the text by the solid state transformation by single crystal to single crystal reaction of **[2–NBD][($BAr^F{}_4$]** with vapors of CH_2Cl_2 to give **[2–NBD][$(CH_2Cl_2)_{0.75}$⊂$BAr^F{}_4$]** the same X-ray data was obtained with similar chemical occupancy for CH_2Cl_2 (freely refined to ~ 0.75, R-factor = 5.1) which resulted with the same molecular structure for **[2–NBD][$(CH_2Cl_2)_{0.75}$⊂$BAr^F{}_4$]** as the one obtained by solution methods.

X-ray crystal structure of [2–NBD][($BAr^F{}_4$] (CCDC 1946575)

Crystal data for **[2–NBD][($BAr^F{}_4$]**: $C_{64}H_{66}BF_{24}P_2Rh$, M = 1466.82 g/mol, monoclinic, $P2_1/c$, a = 13.68100(10), b = 18.61720(10), c = 26.5173(2) Å, β = 93.0740(10)°, V = 6744.29(8) Å^3, Z = 4, λ(Cu-Kα) = 1.54184 Å, T = 150(1) K, orange prism, ρ(calcd, g cm^{-3}) = 1.445, μ (mm^{-1}) = 3.431, 56742 reflections collected, 13979 independent measured reflections (R_{int} = 0.0385), F^2 refinement, R_1(obs, $I > 2\sigma(I)$) =

0.0398, wR_2(all data) = 0.1094, 13002 independent observed reflections [$|F_o| > 4\sigma(|F_o|)$, $2\theta_{max}$ = 152.4°], 640 restrains, 997 parameters, GOF = 1.020 and residual electron density (e Å^{-3}) = 1.216/-0.659.

***Additional details for* [2–NBD][(BAr$^F{}_4$]**: This compound crystallized in the monoclinic space group $P2_1/c$ with one independent molecule in the asymmetric unit. Six CF_3 groups were modelled as disordered. After the solid-state transformation by single crystal to single crystal reaction from **[2–NBD][(BAr$^F{}_4$]** to **[2–NBD][(CH_2Cl_2)$_{0.75}$⊂BAr$^F{}_4$]** no residual electron density was detected in the difference Fourier map which could be attributed to residual amounts of CH_2Cl_2 in the crystal lattice.

X-ray crystal structure of [2–NBD][(Xe)$_{0.5}$⊂BAr$^F{}_4$] (CCDC 1946576)

***Crystal data for* [2–NBD][(Xe)$_{0.5}$⊂BAr$^F{}_4$]**: $C_{64}H_{66}BF_{24}P_2RhXe_{0.5}$, M = 1532.47 g/mol, monoclinic, $P2_1/c$, a = 13.71830(10), b = 18.7287(2), c = 26.5361(2) Å, β = 92.8740(10)°, V = 6809.24(10) Å^3, Z = 4, λ(Cu-Kα) = 1.54184 Å, T = 150(1) K, orange prism, ρ(calcd, g cm^{-3}) = 1.495, μ (mm^{-1}) = 5.313, 40610 reflections collected, 14061 independent measured reflections (R_{int} = 0.0340), F^2 refinement, R_1(obs, $I > 2\sigma(I)$) = 0.0523, wR_2(all data) = 0.1480, 12140 independent observed reflections [$|F_o| > 4\sigma(|F_o|)$, $2\theta_{max}$ = 152.4°], 696 restrains, 978 parameters, GOF = 1.030 and residual electron density (e Å^{-3}) = 1.176/--1.634.

***Additional details for* [2–NBD][(Xe)$_{0.5}$⊂BAr$^F{}_4$]**: This compound crystallized in the monoclinic space group $P2_1/c$ with one independent molecule in the asymmetric unit. Four CF_3 groups were modelled as disordered. After the solid-state transformation by single crystal to single crystal reaction of **[2–NBD][(BAr$^F{}_4$]** with xenon gas (3 bar, 298 K, 1 week) to give **[2–NBD][(Xe)$_{0.5}$⊂BAr$^F{}_4$]** the electron density detected in the difference Fourier map freely refined to a chemical occupancy for xenon of ~ 0.47 (R-factor 5.2). Thus, the chemical occupancy for xenon was set to refine to 0.5 (R-factor = 5.23). For the various periods of reaction time of **[2–NBD][(BAr$^F{}_4$]** with xenon gas (3 bar, 298K, 1 and 3 weeks) the same chemical occupancy for xenon was observed (~ 0.5, R-factor ~ 5). However, no electron density was detected in the difference Fourier map when **[2–NBD][(BAr$^F{}_4$]** was reacted with xenon gas (3 bar, 298 K) for 1 day, thus the molecular structure which resulted from this experiment was unreacted **[2–NBD][(BAr$^F{}_4$]** (R-factor = 4).

SUPPLEMENTARY X-RAY DATA

Molecular structure and metrics of [2–NBD][(CH_2Cl_2)$_{0.75}$⊂BAr$^F_4$]

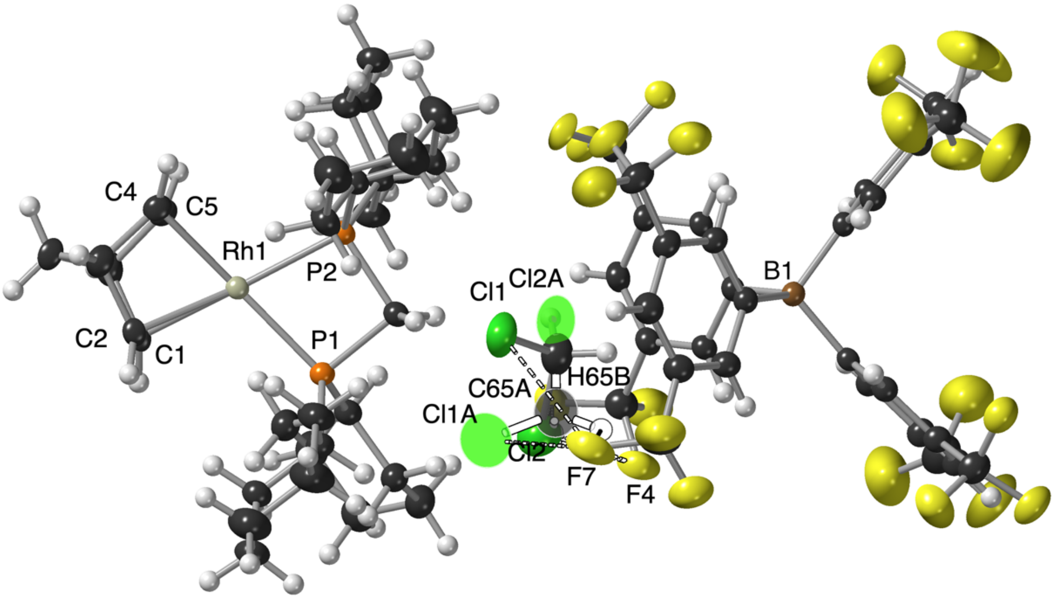

Figure S19. Molecular structure of **[2–NBD][(CH_2Cl_2)$_{0.75}$⊂BAr$^F_4$]** showing the cationic fragment [Rh($Cy_2PCH_2PCy_2$)($\eta^2\eta^2$–NBD)]$^+$ and the anionic [(CH_2Cl_2)$_{0.75}$⊂BAr$^F_4$]$^-$ unit from the asymmetric unit. Displacement ellipsoids displayed at 35% probability. Hydrogen atoms of the phosphine ligand and minor disordered components for CF_3 groups omitted for clarity. Minor disordered component for CH_2Cl_2 ([C65A,Cl1A,Cl2A] chemical occupancy 0.1) pictured as translucent, whereas the major disordered component ([C65,Cl1,Cl2] chemical occupancy 0.65) is pictured as filled ellipsoids) Selected bond lengths (Å) and angles (°): Rh1–P1 2.3009(7), Rh1–P2 2.2888(7), Rh1–C1 2.213(3), Rh1–C2 2.217(3), Rh1–C4 2.207(3), Rh1–C5 2.188(3), C1–C2 1.354(5), C3–C4 1.359(4), P1–Rh1–P2 72.81(3). Pictured with dashed lines CF_3···Cl–CH_2Cl contacts (range 2.685(3)–3.13(2) Å) and CF_3···H_2CCl_2 contacts (range 2.425(2)–3.035(4) Å) between the CH_2Cl_2 molecule and the BAr$^F_4$$^-$ anion in the [(CH_2Cl_2)$_{0.75}$⊂BAr$^F_4$]$^-$ fragment from the asymmetric unit.

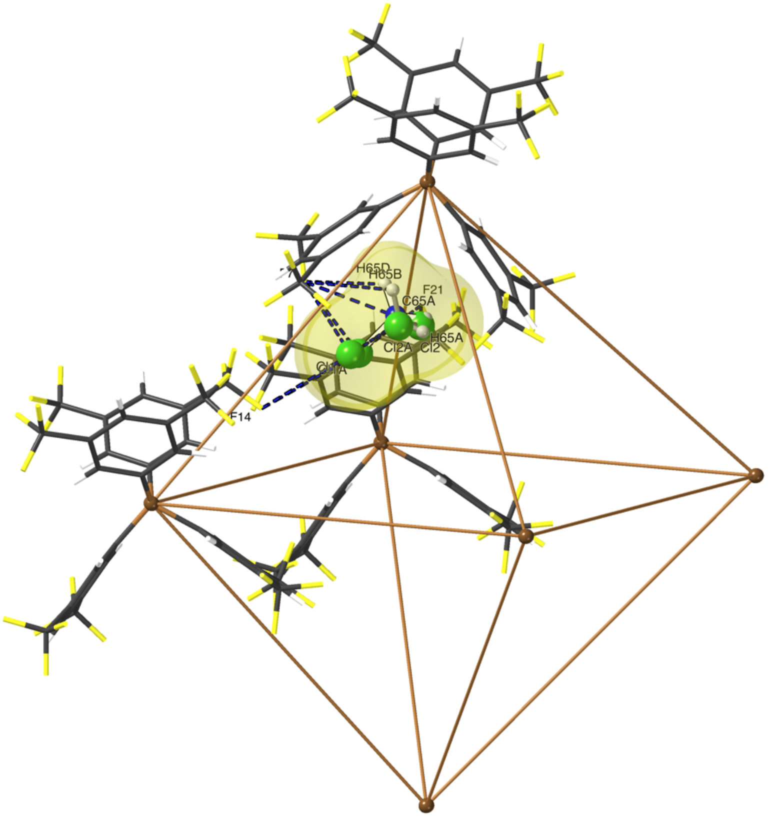

Figure S20. Molecular structure of **[2–NBD][$(CH_2Cl_2)_{0.75}$⊂BAr$^F_4$]** showing the O_h anion BAr$^F{}_4{}^-$ cavity around the encapsulated molecule of CH_2Cl_2. The cationic [Rh($Cy_2PCH_2PCy_2$)($\eta^2\eta^2$–NBD)]$^+$ fragment, non-interacting BAr$^F{}_4{}^-$ anions with the encapsulated CH_2Cl_2 molecule, and minor disordered components the CF_3 groups omitted for simplicity. CH_2Cl_2 molecule pictured in ball and stick, whereas the BAr$^F{}_4{}^-$ anions are shown as sticks for clarity. Pictured with dashed lines $CF_3\cdots Cl–CH_2Cl$ contacts (range 2.685(3)–3.783(4) Å) and $CF_3\cdots H_2CCl_2$ contacts (range 2.425(2)–3.181(3) Å) between the CH_2Cl_2 molecule and the BAr$^F{}_4{}^-$ anions from the O_h cavity.

Molecular structure and metrics of [2–NBD][(BAr$^F_4$]

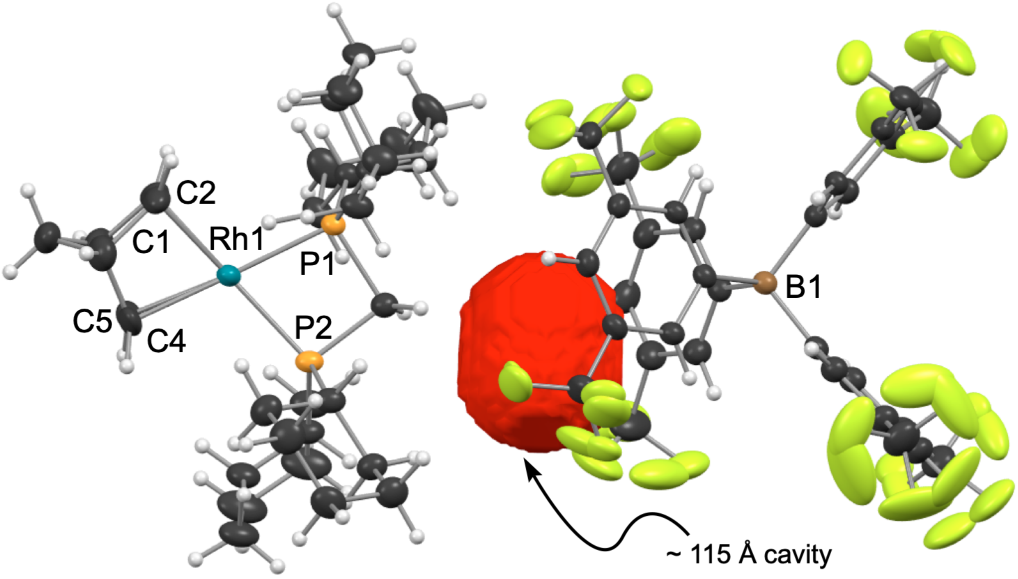

Figure S21. Molecular structure of **[2–NBD][(BAr$^F_4$]** showing the cationic fragment [Rh(Cy$_2$PCH$_2$PCy$_2$)(η^{2}η^2–NBD)]$^+$, the anionic [BAr$^F_4$]$^-$ unit from the asymmetric unit and the cavity. The cavity was calculated using Mercury CSD Software[15] resulting in an empty space of 116 Å^3 (~ 2% of unit cell volume) for a spherical "probe" of radius of 1.5 Å, and a grid spacing of 0.2 Å, which is located between two (CF$_3$)$_2$C$_6$H$_3$ groups from the [BAr$^F_4$]$^-$ anion and the cationic fragment [Rh(Cy$_2$PCH$_2$PCy$_2$)(η^{2}η^2–NBD)]$^+$, same position occupied by the removed CH$_2$Cl$_2$ molecule from **[2–NBD][(CH$_2$Cl$_2$)$_{0.75}$⊂BAr$^F_4$]**. Displacement ellipsoids displayed at 35% probability. Hydrogen atoms of the phosphine ligand and minor disordered components for CF$_3$ groups omitted for clarity. Selected bond lengths (Å) and angles (°): Rh1–P1 2.2961(5), Rh1–P2 2.2802(5), Rh1–C1 2.215(2), Rh1–C2 2.217(3), Rh1–C4 2.195(2), Rh1–C5 2.196(2), C1–C2 1.348(4), C3–C4 1.364(4), P1–Rh1–P2 72.77(2).

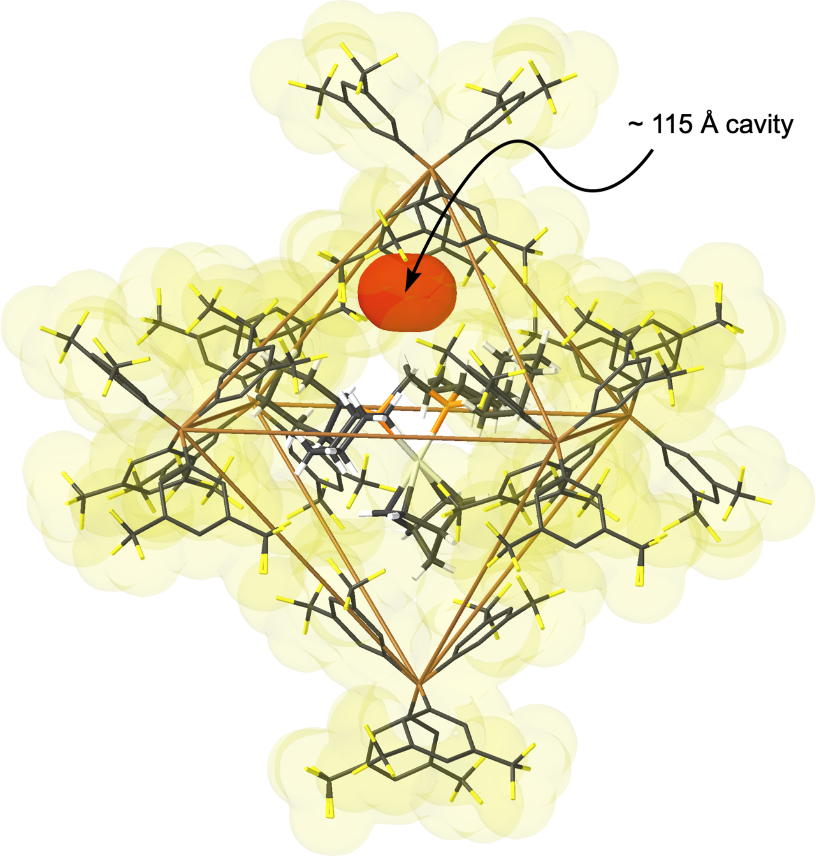

Figure S22. Molecular structure of **[2–NBD][(BAr^F$_4$]** showing the O_h anion BAr^F$_4$$^-$ cavity around the ~ 115 Å cavity[15] resulting from the removal of CH_2Cl_2 from **[2–NBD][(CH_2Cl_2)$_{0.75}$⊂BAr^F$_4$]**. Minor disordered components the CF_3 groups omitted for simplicity. The BAr^F$_4$$^-$ anions and the cationic [Rh($Cy_2PCH_2PCy_2$)($\eta^2\eta^2$–NBD)]$^+$ fragment are pictured as sticks for clarity. This distinct view from the cavity was created with the Crystalmaker software for a spherical "probe" of radius of 1.5 Å.[16]

Molecular structure and metrics of [2–NBD][(Xe)$_{0.5}$⊂BAr$^F_4$]

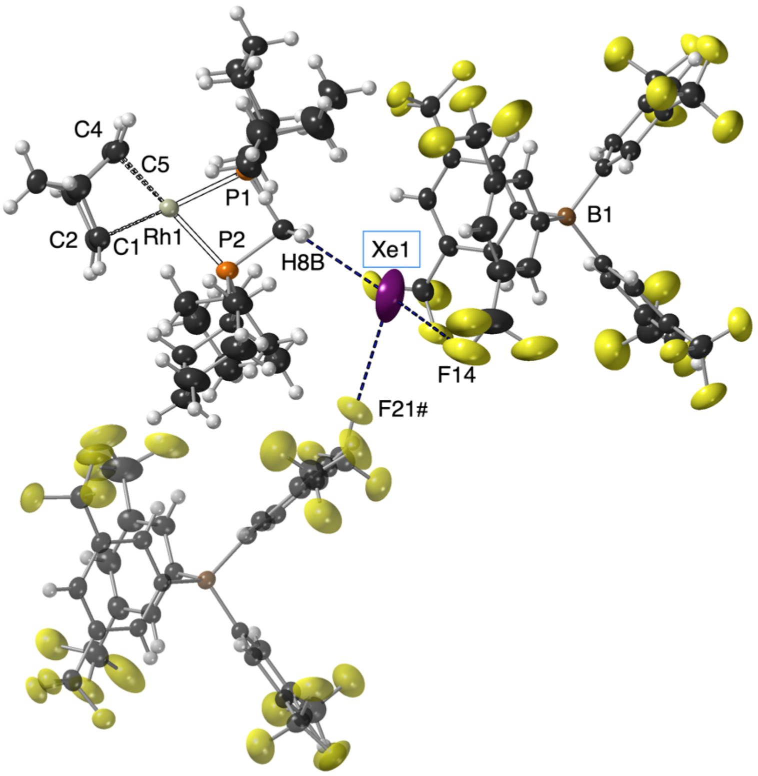

Figure S23. Molecular structure of **[2–NBD][(Xe)$_{0.5}$⊂BAr$^F_4$]** showing the cationic fragment [Rh(Cy$_2$PCH$_2$PCy$_2$)(η^{2}η^2–NBD)]$^+$ and the anionic [(Xe)$_{0.5}$⊂BAr$^F_4$]$^-$ unit from the asymmetric unit showing Xe···FCF$_2$ and Xe···HCHP$_2$ contacts. Displacement ellipsoids displayed at 35% probability. Hydrogen atoms of the phosphine ligand and minor disordered components for CF$_3$ groups omitted for clarity. Selected bond lengths (Å) and angles (°): Rh1–P1 2.2873(8), Rh1–P2 2.2976(8), Rh1–C1 2.214(3), Rh1–C2 2.217(4), Rh1–C4 2.195(4), Rh1–C5 2.200(4), C1–C2 1.361(6), C3–C4 1.355(6), P1–Rh1–P2 72.79(3). Pictured with dashed lines CF$_3$···Xe contacts (F14–Xe1 3.089(9) and F21#–Xe1 3.477(7) Å) between the Xe atom and the BAr$^F_4$$^-$ anion, and Xe···HCHP$_2$ (Xe1···H8B 2.976(1) Å) in the [(Xe)$_{0.5}$⊂BAr$^F_4$]$^-$ fragment. F21# is a symmetry generated atom (–x,1/2+y,1/2–z).

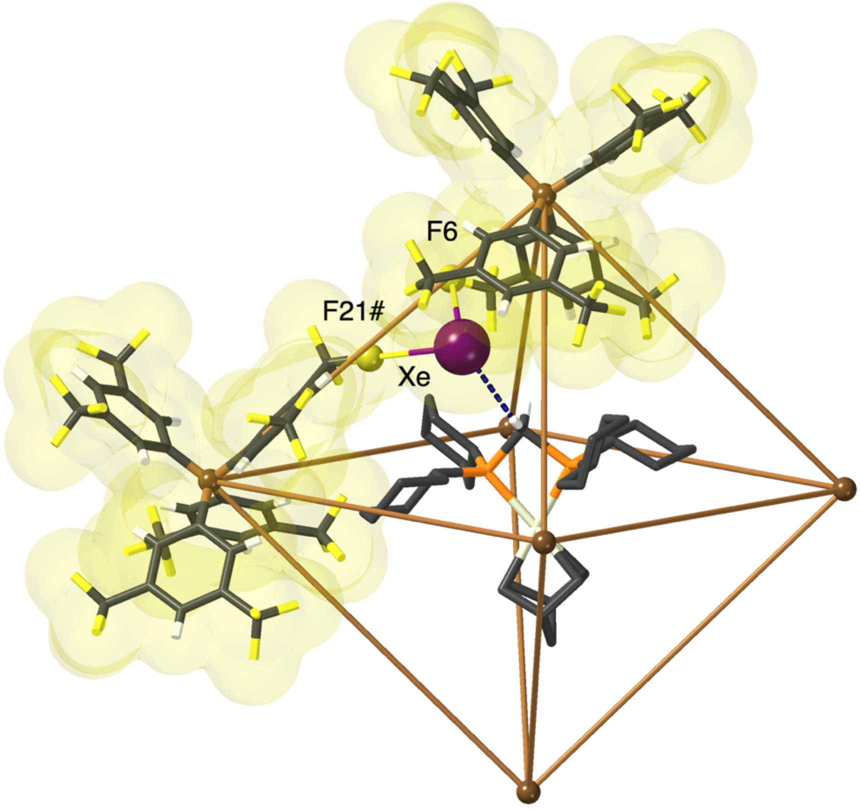

Figure S24. Molecular structure of **[2–NBD][(Xe)$_{0.5}$⊂BAr$^F_4$]** showing the O_h anion BAr$^F_4$$^-$ cavity around the encapsulated Xe. The cationic [Rh($Cy_2PCH_2PCy_2$)($\eta^2\eta^2$–NBD)]$^+$ fragment, non-interacting BAr$^F_4$$^-$ anions with the encapsulated Xe, and minor disordered components the CF_3 groups omitted for simplicity. Xe pictured in ball and stick, whereas the BAr$^F_4$$^-$ anions and the [Rh($Cy_2PCH_2PCy_2$)($\eta^2\eta^2$–NBD)]$^+$ fragment are shown as sticks for clarity. Pictured with dashed lines CF_3···Xe contacts (F14–Xe1 3.089(9), F21#–Xe1 3.477(7) Å) between the Xe atom and the BAr$^F_4$$^-$ anion, and Xe···$HCHP_2$ (Xe1···H8B 2.976(1) Å) in the [(Xe)$_{0.5}$⊂BAr$^F_4$]$^-$ fragment. F21# is a symmetry generated atom (–x,1/2+y,1/2–z).

REFERENCES

[1] B. Guzel, M. A. Omary, J. P. Fackler, A. Akgerman, *Inorg Chim Acta* **2001**, *325*, 45-50.
[2] A. J. Martínez-Martínez, A. S. Weller, *Dalton Trans.* **2019**, *48*, 3551-3554.
[3] A. L. Colebatch, A. I. McKay, N. A. Beattie, S. A. Macgregor, A. S. Weller, *Eur. J. Inorg. Chem.* **2017**, *2017*, 4533-4540.
[4] B. J. van Rossum, H. Förster, H. J. M. de Groot, *J. Magn. Reson.* **1997**, *124*, 516-519.
[5] O. B. Peersen, X. L. Wu, I. Kustanovich, S. O. Smith, *J Magn Reson Ser A* **1993**, *104*, 334-339.
[6] C. J. Jameson, in *Multinuclear NMR* (Ed.: J. Mason), Springer US, Boston, MA, **1987**, pp. 463-477.
[7] W. L. Earl, D. L. Vanderhart, *J. Magn. Reson.* **1982**, *48*, 35-54.
[8] K. Saalwächter, R. Graf, H. W. Spiess, *J. Magn. Reson.* **1999**, *140*, 471-476.
[9] A. T. Lubben, J. S. McIndoe, A. S. Weller, *Organometallics* **2008**, *27*, 3303-3306.
[10] J. Cosier, A. M. Glazer, *J. Appl. Cryst.* **1986**, *19*, 105-107.
[11] J. VandeVondele, J. Hutter, *J. Chem. Phys.* **2007**, *127*, 114105.
[12] G. M. Sheldrick, *Acta Cryst. Sect. A* **2015**, *71*, 3-8.
[13] G. M. Sheldrick, *Acta Cryst. Sect. A* **2008**, *64*, 112-122.
[14] O. V. Dolomanov, L. J. Bourhis, R. J. Gildea, J. A. K. Howard, H. Puschmann, *J. Appl. Cryst.* **2009**, *42*, 339-341.
[15] a) Calculated using the contact surface from the CIF data for **[2–NBD][(BAr$^F_4$]** using Mercury CSD 4.1.3 (Build 249162) for a spherical probe of 1.5 Å, grid spacing 0.2 Å: C. F. Macrae, P. R. Edgington, P. McCabe, E. Pidcock, G. P. Shields, R. Taylor, M. Towler, J. van de Streek, *J. Appl. Cryst.* **2006**, *39*, 453-457; b) L. J. Barbour, *Chem. Comm.* **2006**, 1163-1168.
[16] Palmer, D. C. (2014). CrystalMaker. CrystalMaker Software Ltd, Begbroke, Oxfordshire, England.

AUTHOR CONTRIBUTIONS

The manuscript was written through contributions of all authors. All authors have given approval to the final version of the manuscript.
